# Loss of *putzig* Activity Results in Apoptosis during Wing Imaginal Development in *Drosophila*

**DOI:** 10.1371/journal.pone.0124652

**Published:** 2015-04-20

**Authors:** Mirjam Zimmermann, Sabrina J. Kugler, Adriana Schulz, Anja C. Nagel

**Affiliations:** Institute of Genetics, University of Hohenheim, 70599 Stuttgart, Germany; University of Bern, SWITZERLAND

## Abstract

The *Drosophila* gene *putzig (pzg)* encodes a nuclear protein that is an integral component of the Trf2/Dref complex involved in the transcription of proliferation-related genes. Moreover, Pzg is found in a complex together with the nucleosome remodeling factor NURF, where it promotes Notch target gene activation. Here we show that downregulation of *pzg* activity in the developing wing imaginal discs induces an apoptotic response, accompanied by the induction of the pro-apoptotic gene *reaper*, repression of *Drosophila* inhibitor of apoptosis protein accumulation and the activation of the caspases Drice, Caspase3 and Dcp1. As a further consequence ‘Apoptosis induced Proliferation’ (AiP) and ‘Apoptosis induced Apoptosis’ (AiA) are triggered. As expected, the activity of the stress kinase Jun N-terminal kinase (JNK), proposed to mediate both processes, is ectopically induced in response to *pzg* loss. In addition, the expression of the mitogen *wingless* (*wg*) but not of *decapentaplegic* (*dpp*) is observed. We present evidence that downregulation of *Notch* activates Dcp1 caspase and JNK signaling, however, neither induces ectopic *wg* nor *dpp* expression. In contrast, the consequences of *Dref*-RNAi were largely indistinguishable from *pzg*-RNAi with regard to apoptosis induction. Moreover, overexpression of *Dref* ameliorated the downregulation of *pzg* compatible with the notion that the two are required together to maintain cell and tissue homeostasis in *Drosophila*.

## Introduction

Cellular and tissue homeostasis describes a complex process ensuring the survival and correct development of an organism. Apoptosis, the major form of programmed cell death, contributes to tissue homeostasis by eliminating aberrant, surplus or malignant cells during 'normal' development and in response to stress induced conditions. This safeguarding system demands a fine-tuned control as an unregulated apoptosis has been connected to various human diseases including cancer (reviewed in [1–4]). Research in several model organisms including *Drosophila* has expanded our knowledge on the molecular mechanisms underlying the well conserved apoptotic execution program in metazoans: Under normal conditions, cell survival is guaranteed by the Inhibitors of Apoptosis Proteins (IAP, DIAP1 in *Drosophila*), which bind and inhibit caspases, the key executing enzymes of apoptosis (reviewed in [4–6]). In contrast, if cell death is triggered, e.g. under cellular stress conditions, pro-apoptotic gene activity is induced in *Drosophila*: Pro-apoptotic gene products include the DIAP1-antagonists Hid, Rpr and Grim, which themselves mediate the ubiquitin dependent degradation of DIAP1 thereby enabling caspases to provoke the death of the cell [7–13].

Although diverse stress signals provoke a strong apoptotic answer in the respective tissue, organisms can often compensate this cell loss allowing them to survive with no or only minor consequences on the final tissue or body size. This intriguing fact has been designated Apoptosis-induced Proliferation (AiP) and describes the striking property of dying cells to stimulate proliferation of adjacent surviving cells, and therefore tissue regeneration (reviewed in [14–17]). The model system *Drosophila* with its sophisticated genetic methods offers the opportunity to study the mechanisms underlying the communication between damaged cells and the surrounding tissues. Here, cell death was experimentally induced in cells of larval imaginal discs and, as a consequence, the ectopic induction of mitogens like *wingless* (*wg*), *decapentaplegic* (*dpp*) or *hedgehog* (*hh*) was often observed in the apoptotic cells [18–21]. These mitogens, the key players in the regulation of morphogenesis and growth in the course of *Drosophila* development [22,23], provide a well-founded explanation for the compensatory proliferation of neighboring cells. However, since the genuine dying cells are often rapidly eliminated, much of our current knowledge on AiP was deduced from a special type of apoptotic cells referred to as 'undead' cells. These cells are experimentally obtained by provoking cell death while expressing the caspase inhibitor p35 at the same time. p35 specifically blocks the function of the effector caspases Drice and Dcp-1 without affecting the activity of the initiator caspase Dronc [19,24,25]. Therefore, these cells are trapped in the execution of cell death, but fail to complete the process due to the blocked function of effector caspases [18,19,26]. This experimental approach led to the conclusion that the Jun N-terminal kinase (JNK) acts as a central player of AiP in the dying cells. A robust activity of this stress kinase is associated with diverse aspects of tissue regeneration, including the expression of the aforementioned mitogens and the delay of larval development that keeps the animal in the growth phase ([27–28]; reviewed in [29]). Moreover, as dying cells lose their epithelial integrity, JNK-signaling enforces the restitution of an intact epithelium including the formation of actin cables and filopodia in accordance with its well-defined role in the healing of epidermal wounds [30–33].

Intriguingly, JNK-signaling activity is not only associated with emanating proliferative signals from the apoptotic cells, but also with the non-autonomous induction of secondary cell death at a considerable distance from the primary cell death source. This phenomenon was recently termed Apoptosis induced Apoptosis (AiA) and might be the mechanistic explanation for the systemic cell death occurring both in normal development of metazoans as well as in some human pathologies like e.g. neurodegenerative disorders ([34]; reviewed in [35]). Obviously, tissue homeostasis is coordinated by a cross-regulatory relationship of widespread signaling molecules keeping proliferation and apoptosis in a balanced ratio.

The DNA replication-related element-binding factor (Dref)-complex as well as the Notch (N) signaling pathway are both suitable candidates for being part of such a regulatory network, as they are known to govern numerous developmental processes including cell proliferation and apoptosis (reviewed in [36–38]). Dref acts as a transcription factor in *Drosophila* and is proposed to regulate the expression of a multitude of genes required for cell proliferation such as cell cycle regulators, growth factors or DNA replication factors (reviewed in [37]). A similar pleiotropic influence is mediated by the highly conserved N signaling pathway, being reiteratively used during the development of a variety of tissues in higher eumetazoa. Depending on the cellular context, N can either promote or inhibit growth processes emphasizing the importance of a tight and fine-tuned regulation of the signaling cascade (reviewed in [38,39]). In *Drosophila*, both signaling networks depend on *putzig* (*pzg*), an essential positive regulator of the Dref and N signaling cascades [40,41]. Pzg is an integral component of the Trf2/Dref protein complex that regulates proliferation-related genes [40]. Moreover, Pzg acts Dref independently and promotes N target gene activation via the Nucleosome remodeling factor (Nurf), implying a Pzg-mediated epigenetic influence on N target gene activation [41].

Here, we show that reduction of *pzg* activity during larval wing development results in the induction of genuine dying cells which initiate AiP mechanisms and enable AiA. As expected, ectopic JNK-signaling activity is induced autonomously and non-autonomously, likely to mediate the systemic response. This spectrum of consequences is not mimicked by a downregulation of *N* receptor activity: Though apoptosis and proliferation are induced, the latter is not mediated by the ectopic induction of *wg* in the genuine dying cells, unlike in the *pzg* depleted cells. In contrast, a downregulation of *Dref* activity does not only provoke apoptosis but also AiP, mediated by the induction of *wg*, similar to the effects observed after *pzg* knockdown. We conclude that Pzg is fundamental for the fine-tuned homeostasis of cell survival and proliferation via its influence on important signaling networks during the development of *Drosophila*.

## Materials and Methods

### Fly stocks, genetics and work

The following fly stocks were used:

#### Gal4/UAS lines

EP-*pzg* (EP-*756*; Exelixis stock collection, USA). Gal4-lines: *en-*Gal4 UAS-*GFP* [42]; *en-*Gal4 UAS-*GFP* UAS*-pzg-*RNAi*/*CyO [40]; *Gmr-*Gal4 [43]; *Gmr-grim/*TM3Sb [9]; *Gmr-hid/*CyO [8]; *Gmr-rpr/*TM6B [44]; *Omb* ^*md65*^ *-*Gal4*/*FM7 [45]. UAS-lines: UAS-*Dref* [46]; gift from D. Bohmann); UAS-*Dref*-RNAi (BL31941); UAS*-lacZ* (BL8529); UAS-*H*-RNAi [47]; UAS-*N*-RNAi (BL7078); UAS*-p35* (BL6298); UAS-*pzg*-RNAi [40]; UAS-*pzg*-RNAi (VDRC v25542).

#### Reporter-strains

*dpp*-lacZ [48]; *puc-*lacZ [49]; *rpr-*lacZ [50].

Flies and crosses were raised on standard fly food supplemented with fresh yeast paste at 25°C. Crosses with UAS-RNAi lines were cultured at 29°C to ensure strong RNAi induction.

### Antibody staining and documentation with confocal microscopy

Antibody staining on wing imaginal discs was done according to Müller *et al*. [51]. The following antibodies were used: guinea-pig anti-Hairless (H) (1:500) [52]; guinea-pig anti-Pzg (1:500) [40]; mouse anti-Arm (1:50; DSHB); mouse anti-beta Galactosidase (1:50; DSHB); mouse anti-Notch (ICN) (1:25; DSHB); mouse anti-Wg (1:50; DSHB) all obtained from the Developmental Studies Hybridoma Bank, Department of Biological Science, University of Iowa City, IA 52242, USA; mouse anti-DIAP1 (1:400) [13]; rabbit anti-activated Caspase 3 (1:200; Cell Signaling, Germany); rabbit anti-activated Dcp-1 (1:200; Cell Signaling, Germany), rabbit anti-activated Drice (1:250 [13]; rabbit anti-GFP (1:100; Santa Cruz, USA); rabbit anti-Phospho-Histone H3 (PH3) (1:50; Cell Signaling, Germany); rat anti-Dilp8 (1:500 [53]; gift from P. Léopold, Nice, France). Secondary antibodies coupled to fluorescein, Cy3 or Cy5 were purchased from Jackson Laboratories (Dianova, Germany). Dissected tissues were mounted in Vectashield (Vector Laboratories, USA).

For labeling cells undergoing DNA synthesis, the Click-iT EdU Alexa Fluor 488 Imaging Kit (Invitrogen, Eugene, Orgeon, USA) was used. After dissection in ice-cold PBS, larval tissues were incubated for two hours at room temperature in M3 insect medium including 10 mM EdU stock solution. A 4% para-formaldehyde fixative was added for 25 minutes, removed, and washed at least four times with PBS, followed by a 20 minutes incubation with PBX (PBS with 0.3% Triton X-100) and two washing steps for 10 minutes each. The Click-iT reaction cocktail was added containing 443.5 μl H_2_O, 21.5 μl 10X Click-iT reaction buffer, 10 μl CuSO_4_, 0.5 μl Alexa Fluor Azide and 25 μl reaction buffer additive, and the discs were incubated for 30 minutes in the dark. After removal of the reaction cocktail solution, the tissues were washed with PBS two times, prepared and embedded in Vectashield (Vector Laboratories, USA).

Confocal images were acquired with a Zeiss Axioskop linked to a Bio-Rad MRC1024 scanhead by using Bio-Rad Laser Sharp 3.1 software.

### Documentation of adult eyes and statistical quantification of eye size

Adult eyes of females were documented with an ES120 camera (Optronics, Goleta CA, USA) using Pixera viewfinder software version 2.0. For quantification, eye size of five females each was measured with Image J and eye area was calculated. Statistical significance of probes was determined according to Student's T-test (http://www.physics.csbsju.edu/stats/t-test.html) and p-value was scaled accordingly: p>0.05 (not significant, n.s.); p<0.05 (weakly significant; *); p<0.01 (significant; **); p<0.001 (highly significant; ***).

## Results

### *Pzg* genetically interacts with pro-apoptotic genes and is required for cell survival

**Fig 1:**
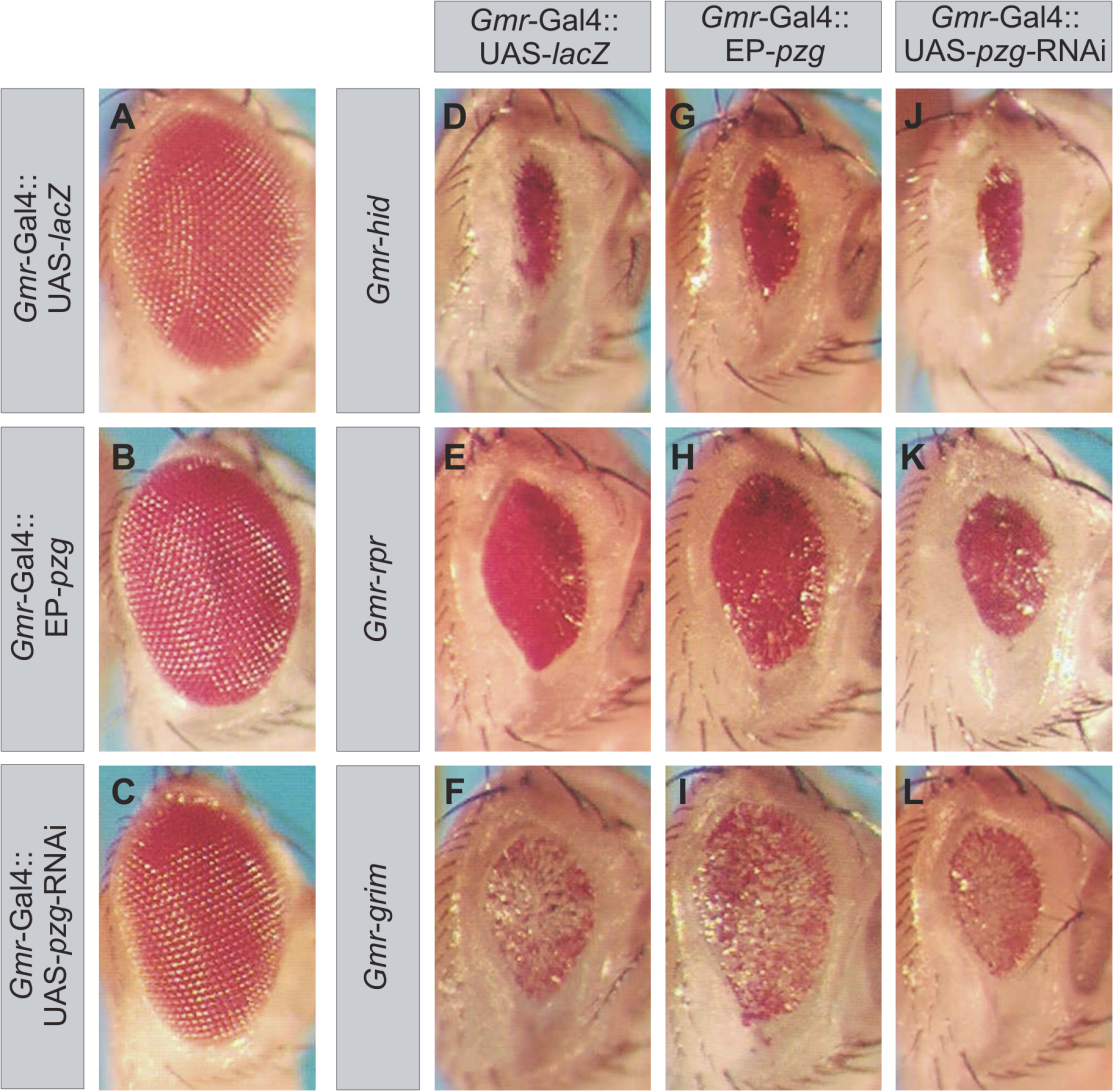
*pzg* genetically interacts with pro-apoptotic genes. (**A-C**) Control eyes after overexpression of *lacZ* and *pzg*, as well as *pzg-*RNAi induction. (**D-F**) Overexpression of *hid*, *rpr* and *grim* during eye development causes small and rough eyes in the adults. (**G-I**) Overexpression of *pzg* ameliorates the small eye size resulting from ectopically expressed pro-apoptotic genes, whereas depletion of *pzg* activity by RNAi enhances the phenotypes (**J-L**). Genotypes analyzed: *Gmr*-Gal4/+; UAS-*lacZ/+*. *Gmr*-Gal4/+; EP-*pzg*/+. *Gmr*-Gal4/+; UAS-*pzg-*RNAi/*+*. *Gmr-hid*/*Gmr*-Gal4; UAS-*lacZ*/+. *Gmr*-Gal4/+; *Gmr-rpr* or *Gmr-grim/* UAS-*lacZ*. *Gmr-hid*/*Gmr*-Gal4; EP-*pzg*/+. *Gmr*-Gal4/+; *Gmr-rpr* or *Gmr-grim*/EP-pzg. *Gmr-hid*/*Gmr*-Gal4; UAS-*pzg-*RNAi/*+*. *Gmr*-Gal4/+; *Gmr-rpr* or *Gmr-grim*/ UAS-*pzg-*RNAi.

Knock down of *pzg* gene activity by *pzg*-RNAi induction during larval development results in tissue size reduction (Fig 1A–1C). Previously, we have shown that *pzg*-RNAi effects are negatively correlated with cell cycle progression, thereby influencing cell proliferation and cell growth [40]. In order to test whether tissue loss and size reduction might also be a consequence of the induction of programmed cell death, we performed genetic interaction assays with the well defined apoptosis-inducing factors *hid*, *rpr* and *grim*, whose transcriptional activation is an essential step in the execution of most apoptotic events in the development of *Drosophila* [54–57] (reviewed in [58]). It is well established that ectopic expression of pro-apoptotic genes in the developing eye imaginal disc using the *Gmr* promoter causes cell killing and therefore conspicuously small eyes in the adults (Fig 1D–1F). Eye size reduction was considerably suppressed by additional expression of Pzg (Fig 1G–1I and S1 Fig), whereas it was enhanced by *pzg-*RNAi induction within the affected tissue (Fig 1J–1L and S1 Fig).

To address this phenomenon in more detail, we examined the expression of several components of the cell death machinery in wing imaginal discs upon *pzg* depletion. We have chosen the wing disc tissue for our analyses, as there the endogenous level of apoptosis is comparatively low [18,59]. Thus higher levels of apoptosis can be attributed to a reduced *pzg* activity.

**Fig 2:**
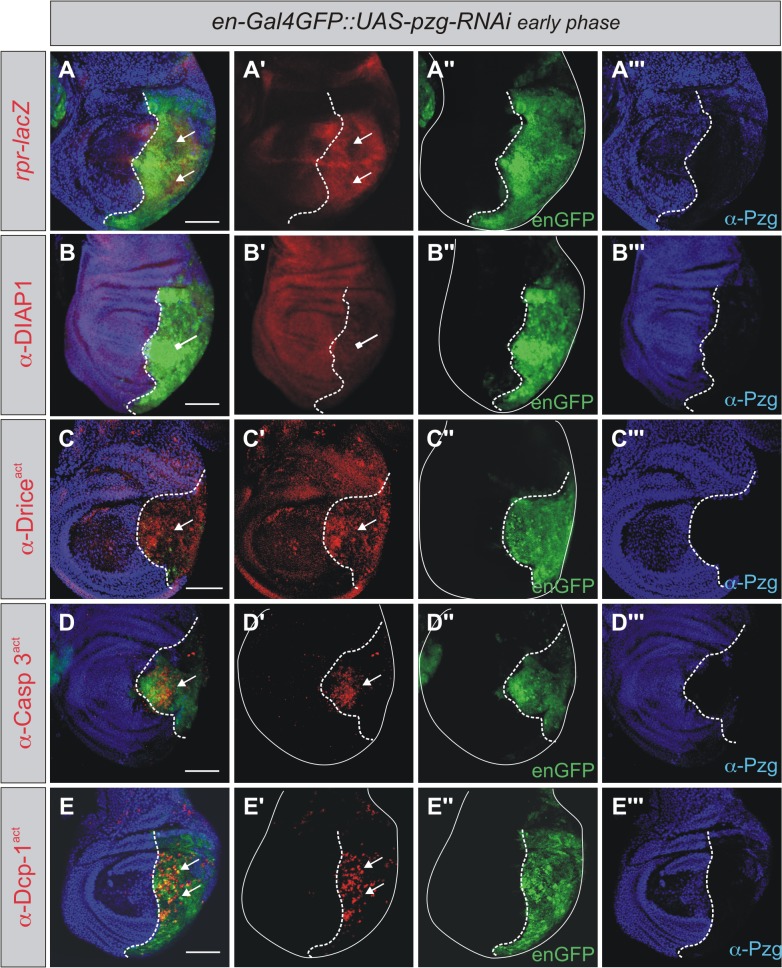
*pzg* depletion autonomously triggers the apoptotic signaling cascade in wing imaginal discs. RNAi mediated depletion of *pzg* was induced in the posterior part of the wing disc using *en*-Gal4. *Rpr*-lacZ, DIAP1 and caspase activity (in red) was monitored as indicated. **(A-A''')** A strong activation of the pro-apoptotic gene *rpr* (arrows) as well as the *Drosophila* activated caspases Drice^act^ (**C-C'''**, arrow), Caspase 3^act^ (**D-D'''**, arrow) and Dcp-1^act^ (**E-E'''**, arrows*)* is detected in the posterior half of the disc, whereas DIAP1 protein level is reduced (**B-B'''**, repressive arrow). (A-A''') *en-*Gal4 UAS-*GFP* UAS*-pzg-*RNAi/*+*; *rpr-*lacZ*/+*. (B-E''') *en-*Gal4 UAS-*GFP* UAS*-pzg-*RNAi/*+*. Pzg protein is shown in blue (anti-Pzg, A-E and A'''- E'''); GFP in green (*en-Gal4 GFP*) marks the posterior compartment (A-E and A''-E''). Posterior is to the right and dorsal up. The antero-posterior compartment boundary is marked with a dashed line. Scale bars: 100 μm.

*Pzg* activity was downregulated with the UAS-*pzg*-RNAi shown before to efficiently reduce Pzg protein levels [40]. *Pzg*-RNAi induction either in the posterior compartment (using *en*-Gal4) or in a more central area (using *omb*-Gal4) of the wing disc triggered the execution of the apoptotic program: Pro-apoptotic gene activity, visualized by the activation of a *rpr*-lacZ reporter (Fig 2A-A''' and S2A-A'' Fig), a reduced level of the anti-apoptotic protein DIAP1 (Fig 2B-B''' and S2B-B'' Fig) and finally the accumulation of activated initiator and effector caspases Drice, Caspase-3 and Dcp-1 were observed in the *pzg* mutant area of the wing disc (Fig 2C-E''' and S2C-E'' Fig). To exclude, however, off-target effects, a second independent RNAi-line (VDRC v25542) was included in the analysis of apoptosis induction and gave the same overall results. These data show that an impaired *pzg* activity leads to an increase in apoptosis.

### *pzg* silencing triggers JNK-mediated stress responses

**Fig 3:**
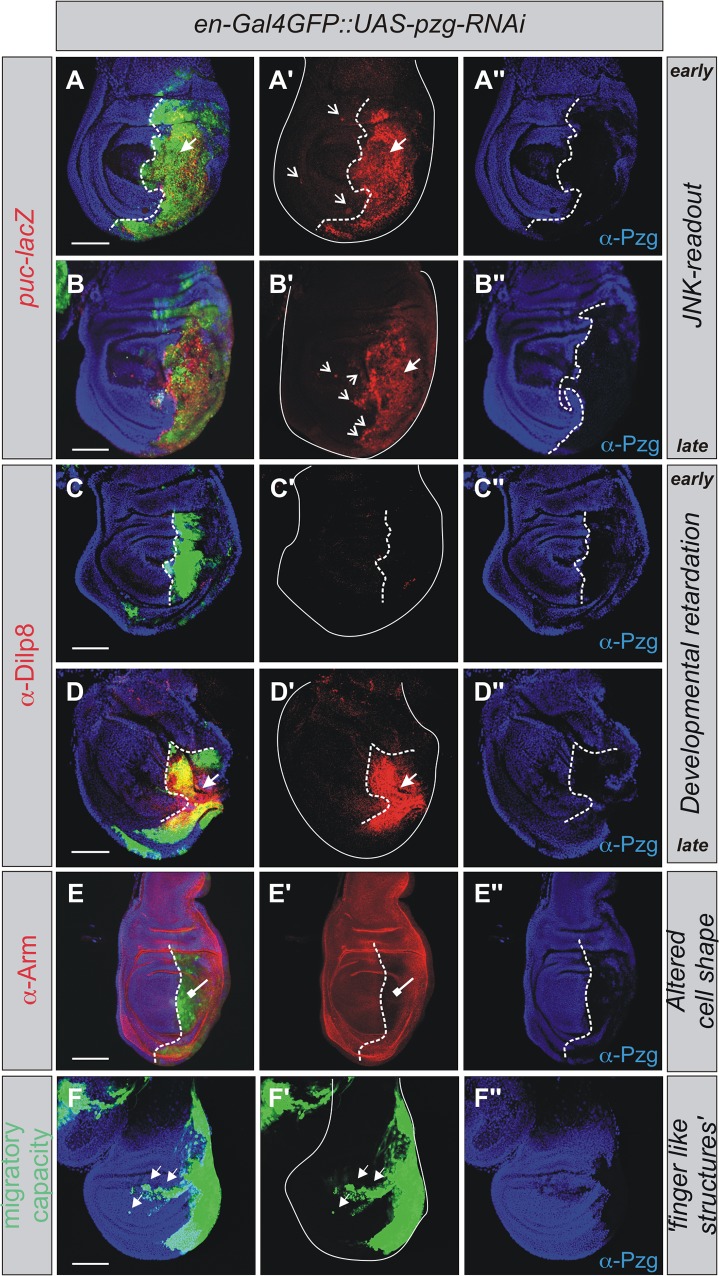
*pzg* depletion results in an inappropriate JNK-signaling activation. *pzg* was downregulated in the posterior part of the wing disc (Pzg protein is shown in blue). (**A-B''**) *puc*-lacZ activity is induced in *pzg*-RNAi mutant cells reflecting JNK-signaling activity (red in A-A', B-B', arrows) (*en-*Gal4 UAS-*GFP* UAS*-pzg-*RNAi*/+; puc-*lacZ*/+)*. In addition, especially in late third instar larval discs, a non-autonomous activity can be detected in the anterior compartment (open arrows in A', B'). (**C-F''**) Further consequences of JNK-mediated developmental apoptosis induction can be observed: (**C-D''**) Dilp8 protein is secreted in the *pzg* depleted cells, however not before late third larval instar (red in D, D', arrow). (**E-E''**) In *pzg*-RNAi mutant cells, Arm protein accumulation is disturbed (red, repressive arrow). (**F-F''**) *pzg* mutant cells penetrate into the anterior compartment while retaining their posterior identity (green, arrows in F, F'). (C-F'') *en-*Gal4 UAS-*GFP* UAS*-pzg-*RNAi*/+*. The A/P compartment boundary is marked with a dotted line. Scale bars: 100 μm.

A key factor known to convey apoptosis in *Drosophila* and mammals alike is the c-Jun N-terminal kinase (JNK) pathway (reviewed in [60,61]). To measure JNK signaling activity in *pzg* mutant cells, we made use of the JNK downstream effector *puckered* (*puc*) and monitored the expression of a *puc*-lacZ enhancer trap line [62]. In wild type third instar wing discs, *puc*-lacZ expression is detected in the small stalk region attaching the disc to the larval epidermis [62]. Knock down of *pzg* robustly activated the *puc*-lacZ reporter gene within the depleted area and weakly in adjacent regions notably in older wing discs (Fig 3A-B'' and S2F-F'' Fig), consistent with the induction of JNK-mediated cell death. Great experimental insights were gained in the recent years demonstrating that JNK-mediated cell death in *Drosophila* is crucial for eliminating aberrant cells, thereby ensuring further development and morphogenesis (reviewed in [16,29,61]). This includes also the induction of a developmental delay and inhibition of metamorphosis, allowing the larva to compensate growth deficits and repair injured tissue. The gene encoding Dilp8, a member of the insulin-relaxin peptide family, was found to be upregulated in response to JNK signaling and is thought to delay metamorphosis by inhibiting ecdysone biosynthesis [53,63]. Tracing Dilp8 in wing discs, where *pzg*-RNAi was induced in the posterior half, revealed no obvious accumulation in younger discs (approximately 96 h AEL; Fig 3C-C''), whereas Dilp8 was highly enriched in later stages (approximately 120 h AEL; Fig 3D-D'').

Interestingly, the older *pzg* mutant wing discs appeared more crumpled and folded than the younger ones (compare Fig 3A and 3C with 3B and 3D). This phenomenon is reminiscent of regenerative growth during larval development experimentally induced with non-surgical tissue damage, e.g. pro-apoptotic gene activity ([64–65]; reviewed in [66]). Moreover it has been shown that cell-adhesion is reduced by the caspase dependent cleavage of Armadillo (Arm) [67,68]. Indeed, we observed a strong reduction of membrane-associated Armadillo (Arm, beta-Catenin) in the *pzg* silenced area of the wing disc (Fig 3E-E''). In a wild type wing disc, the antero-posterior compartment border is straight and defined. In contrast we observed ‘finger-like’ structures notably in older *pzg*-silenced discs (Fig 3F-F''). Apparently, *pzg*-depletion enabled the mutant cells to invade the anterior compartment while still retaining posterior identity (Fig 3F-F'').

### Loss of *pzg* activity results in 'genuine' dying cells and consequently AiP

**Fig 4:**
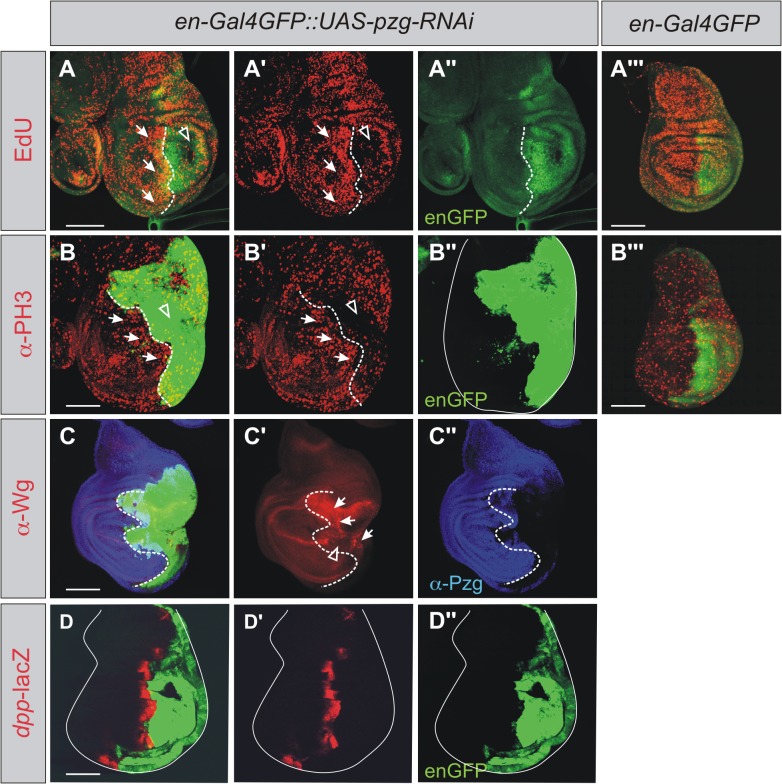
*pzg* mutant cells show characteristics of genuine dying cells and induce AiP. *pzg* was downregulated in the posterior part of the wing disc. Pzg protein is shown in blue (C, C''), the posterior compartment is marked in green with GFP (A-D; A''-D''; A'''-B'''). (**A-A'''**) DNA-synthesis visualized with EdU-labeling (red) is amplified anteriorly along the A/P compartment boundary (arrows) upon *pzg* depletion (A-A') compared to *en-*Gal4 UAS-*GFP* control (A'''), whereas a loss can be observed within the posterior compartment (open arrow, A-A'). (**B-B'''**) Cell division was visualized with anti-PH3 (red). Compared to the control (B'''), many cells in the posterior domain lost this marker upon *pzg* depletion (arrowhead, B-B'), whereas a strip of cells anterior to the A/P boundary shows stronger PH3 labeling (arrows, B-B'). (**C-C''**) Expression of Wg protein was monitored (red): it is interrupted at the dorso-ventral boundary in response to *pzg*-RNAi depletion (arrowhead in C'), whereas ectopic induction of Wg is observed in the posterior compartment outside the normal Wg expression domain (arrows, C'). (**D-D''**) *dpp*-lacZ expression (red) is unchanged by *pzg* depletion in the posterior compartment. Genotypes: (A'''-B''') *en-*Gal4 UAS-*GFP*/+. (A-C'') *en-*Gal4 UAS-*GFP* UAS*-pzg-*RNAi/+. (D-D'') *en-*Gal4 UAS-*GFP* UAS*-pzg-*RNAi*/*+; *dpp*-lacZ/+. Wing discs are oriented posterior rightwards and dorsally upwards. The A/P compartment boundary is marked with a dotted line. Scale bars represent: (A, B, D) 100 μm, (C) 50 μm.

Genetic studies in *Drosophila* have shown that different mechanisms are triggered in apoptotic cells leading to an increase in proliferation and cell division rates of adjacent cells, a process which is referred to as Apoptosis induced Proliferation (AiP) (reviewed in [14,17,69,70]). In order to investigate the consequences of *pzg* depletion with regard to AiP, we monitored the proliferation rates in wing discs, where *pzg*-RNAi was induced in the posterior compartment. As *pzg* was shown to be required for the activation of cell cycle related genes [40], the autonomous decrease of actively dividing cells upon *pzg* depletion compared with controls was expected (Fig 4A-B'''). In addition we noted an elevated number of cells undergoing DNA synthesis (EdU labeled) as well of mitotic cells (PH3 labeled) abutting the *pzg*-RNAi mutant territory at the anterior, implying the induction of a non-autonomous cell division response (Fig 4A-B''').

Apoptotic cells ectopically activate morphogenetic genes like *wingless* (*wg*) or *decapentaplegic* (*dpp*), responsible for the proliferation stimulus in directly adjacent cells [18,19]. To test if this is also a response to *pzg* loss, we firstly analyzed the distribution of Wg protein in wing discs, where *pzg*-RNAi was induced in the posterior compartment. At later stages (approximately 120 h AEL) we observed an ectopic expression of Wg in areas of the posterior compartment (Fig 4C', arrows), whereas the wild type expression along the dorso-ventral boundary in the posterior compartment of the disc was interrupted (Fig 4C', arrowhead). As *wg* is a well known target of N signaling, a reduction of wild type Wg accumulation along the dorso-ventral boundary was expected [40]. Intriguingly, no ectopic induction of *dpp* was observed in *pzg* silenced cells, inferred from the normal expression levels of a *dpp*-lacZ reporter along the antero-posterior compartment boundary (Fig 4D-D''). This demonstrates that *pzg* mutant cells behave like 'genuine' apoptotic cells with respect to the induction of ectopic *wg*, and that they induce AiP without the involvement of ectopic *dpp* activity.

### Loss of *pzg* activity is accompanied by Apoptosis induced Apoptosis (AiA)

**Fig 5:**
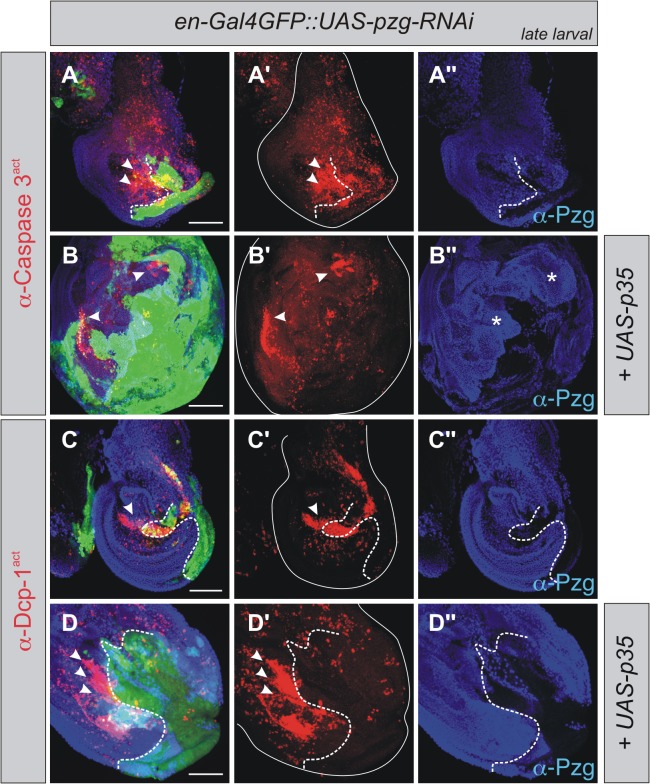
*pzg*-RNAi induces AiA in late larval stages. **(A-D'')** Strong caspase activity, either visualized with anti-Caspase 3^act^ (red in A-A', B-B') or anti-Dcp-1^act^ (red in C-C', D-D'), can be detected non-autonomously in late larval wing disc in the anterior compartment (arrowheads in A-D'). Preventing cell death execution with p35 amplifies the overgrowth effect (asterisks in B'') but still induces AiA in the anterior half of the disc (B-B'', D-D'', arrowheads). Genotypes: (A-A'') and (C-C'') *en-*Gal4 UAS-*GFP UAS-pzg-*RNAi/+. (B-B'') and (D-D'') UAS-*p35; en-*Gal4 UAS-*GFP UAS-pzg-*RNAi/+. The A/P compartment boundary is marked with a dotted line. Scale bars: 100 μm.

Interestingly, cells doomed to die not only trigger AiP but also induce non-autonomous secondary apoptosis, abbreviated AiA (Apoptosis induced Apoptosis) [34]. To examine if such an effect is also observed in *pzg*-RNAi mutant cells we followed the activation of Caspase3 and Dcp1 in late third instar larval wing discs. Under these conditions, a strong caspase activity was observed in the anterior half of the disc, either visualized by staining for cleaved Caspase-3 or Dcp-1 (Fig 5A-A'' and 5C-C''). The co-expression of the baculovirus caspase inhibitor *p35* in the *pzg*-RNAi mutant cells strongly enhanced this effect and triggered tumorous overgrowth of the wing discs and non autonomous AiA (Fig 5B-B'' and 5D-D''). This can be explained by the induction of the apoptotic machinery but the prevention of execution through inhibition of effector caspases by p35, resulting in so-called 'undead' cells [18,19,26,71]. Altogether, these data indicate that loss of *pzg* activity results in apoptosis followed by AiP and AiA.

### Apoptotic consequences of N depletion in wing discs

**Fig 6:**
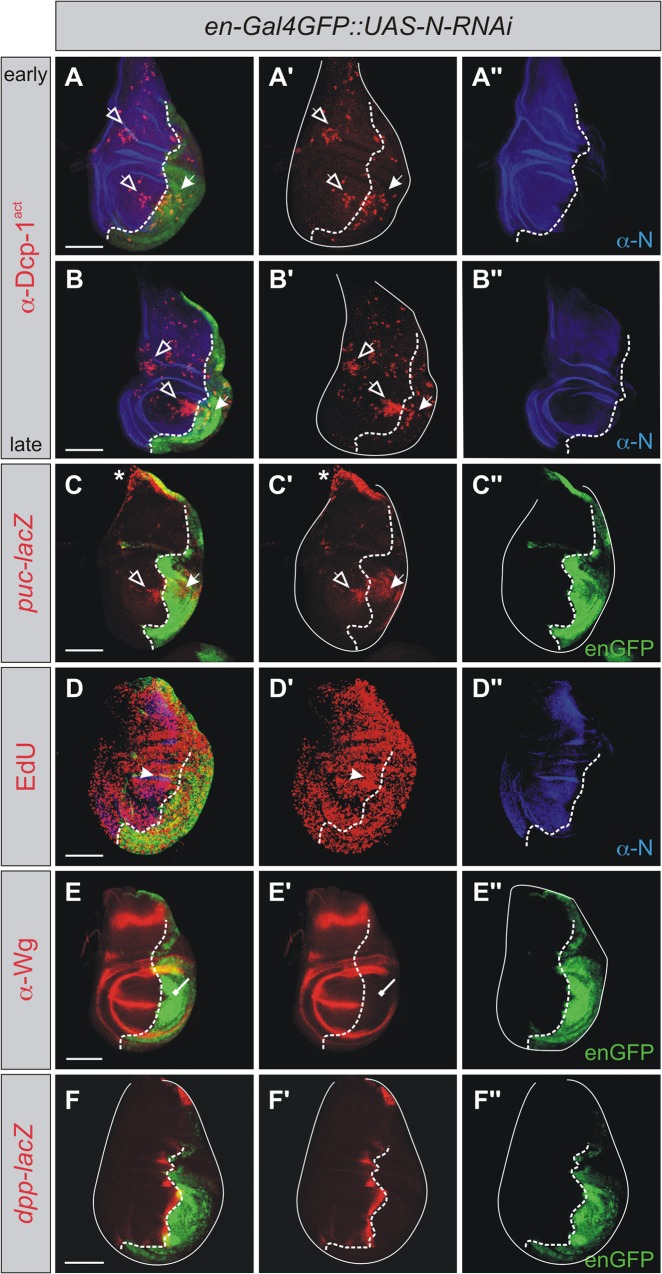
Apoptotic consequences of Notch depletion in wing imaginal discs. *N*-RNAi was induced in the posterior compartment of the wing disc, N protein is shown in blue (A, B, D and A'', B'', D''), the posterior compartment is marked in green with GFP (A-F and C'', E'', F''). (**A-B''**) Caspase activity, visualized with anti-Dcp-1act (red in A, A' and B, B') is detectable in early (96 h AEL) and late third instar wing discs (120 h AEL) autonomously (arrows in A, A' and B, B') and non-autonomously (open arrows in A, A' and B, B'). The effect of AiA is enhanced in later phases of development (B, B'). (Genotype: UAS-*N*-RNAi; *en-*Gal4 UAS-*GFP/+*). (**C-C''**) JNK-signaling readout visualized with *puc*-lacZ expression is seen in both compartments (red in C, C' arrows; UAS-*N*-RNAi; *en-*Gal4 UAS-*GFP/+; puc-*lacZ*/+*). The wild type expression of *puc*-lacZ at the stalk region is marked with an asterisk (C, C'). (**D-D''**) Cells within S-phase are labeled with EdU (red); a minor autonomous reduction and weak increase in most central cells abutting the A/P compartment boundary is observed (arrow in D, D'; genotype as in A). (**E-E''**) The expression of Wg is lost at the dorso-ventral boundary in the posterior compartment (red in E, E', repressive arrow; genotype as in A). (**F-F''**) *dpp* expression is not affected by downregulation of *N* in the posterior compartment (red in F, F'; UAS-*N*-RNAi; *en-*Gal4 UAS-*GFP/*+; *dpp*-lacZ/+). The A/P compartment boundary is marked with a dotted line. Scale bars: 100 μm.

As Pzg was shown to be required for efficient N signaling to occur, we asked whether the observed apoptotic outcome after *pzg*-RNAi induction might be the consequence of an impaired N signaling activity [40,41]. It is well established that a reduction in N signaling activity during imaginal development is correlated with tissue loss and apoptosis [51,72]. Therefore, we induced *N*-RNAi in the posterior compartment of the wing disc to compare the effects with those obtained after *pzg* depletion. To this end, we used the same *N*-RNAi line that has been shown by others to provide RNAi-mediated knock-down of N activity in wing imaginal discs [38,73]. We observed an accumulation of activated Dcp-1 caspase autonomously in *N* mutant cells and also non-autonomously in the anterior compartment, indicating that a downregulation of N signaling contributes to AiA (Fig 6A-B''). Moreover, a robust induction of *puc*-lacZ was detected in both compartments, indicating that JNK-mediated activity was induced as well (Fig 6C-C''). In contrast, cell proliferation, visualized with EdU labeling, was different from *pzg*-RNAi depleted cells: Cells within the *N*-RNAi depleted compartment were still able to cycle through the cell cycle concluded from EdU incorporation (Fig 6D-D''). Based on the modest reduction of EdU signals, less cells however, appeared to enter the S-phase. Moreover, AiP was only weakly observed as EdU labeled cells abutting the *N*-deficient area appeared only more densely spaced (Fig 6D-D''), and no ectopic induction of the mitogens Wg (Fig 6E-E'') or *dpp*-lacZ (Fig 6F-F'') was detected.

In order to investigate, whether an upregulation of N signaling activity could ameliorate the apoptotic consequences of *pzg* loss, we initially sought to overexpress the activated form of the N receptor in *pzg*-RNAi depleted cells. However probably due to the immensely hyper-activated N response, we failed to obtain any viable larvae of this genotype. A more gentle method to increase N signaling activity is to reduce the activity of the general antagonist Hairless (H), e.g. by inducing a *H-*RNAi construct proven to reduce *H* activity in the fly [47]. Reduction of *H* activity in *pzg*-RNAi mutant cells of the wing disc did not considerably abrogate the *pzg*-RNAi induced apoptosis defects (S3 Fig). These data suggest that the cell death resulting from loss of *pzg* is not primarily triggered by an inappropriate N signaling activity.

### Apoptotic consequences of Dref depletion in wing discs

**Fig 7:**
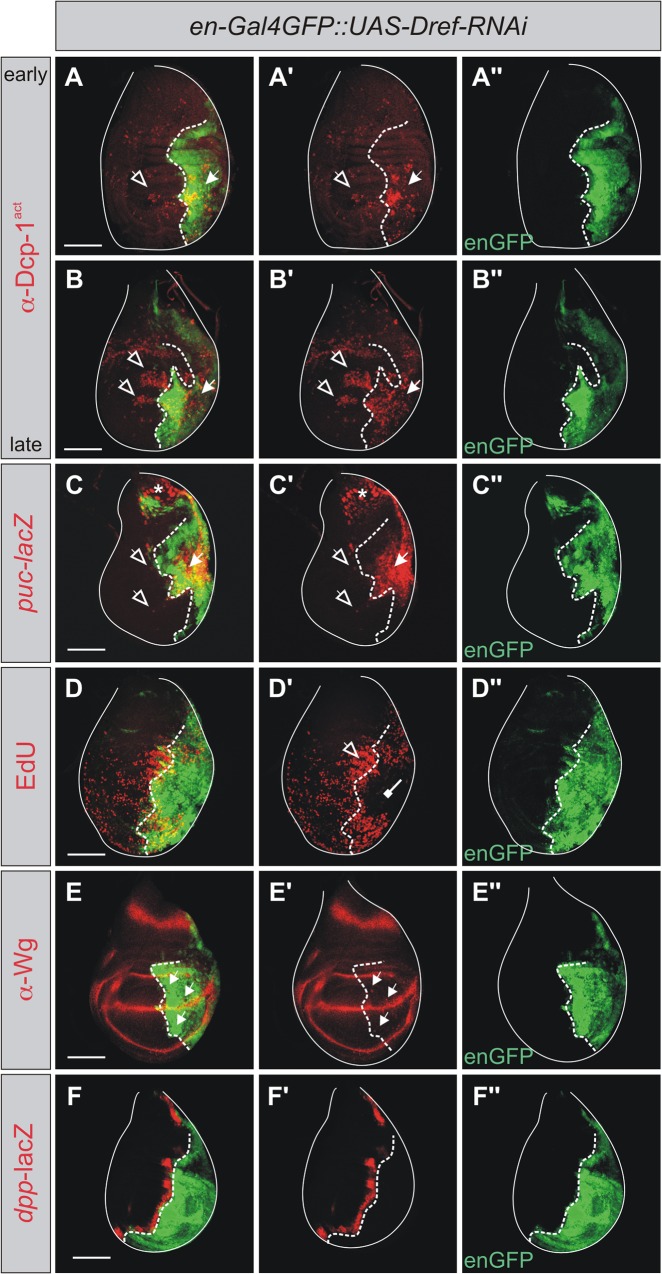
Loss of *Dref* activity entails cell cycle arrest, cell death and AiP. *Dref* was downregulated in the posterior compartment of the wing disc, which is marked in green with GFP (A-F; A''-F''). **(A-B'')** *Dref*-RNAi induction in the posterior compartment of the wing disc is correlated with an autonomous (arrow) and non-autonomous (open arrow) cell death induction, monitored with anti-Dcp1^act^ (red in A, A' and B, B'). Although AiA can be detected already in early third instar wing discs (96 h AEL, A' open arrow), the amount of non-autonomous cell death is strongly increased in later stages (120 h AEL, B' open arrows). (**C-C'')** *puc*-lacZ is ectopically induced in both compartments (red in C, C'; closed and open arrows). Asterisk highlights the wild type expression in the stalk region of the disc. (**D-D''**) Replication is disturbed autonomously after *Dref*-RNAi depletion, visualized with EdU staining (red in D, D', repressive arrow), whereas enhanced proliferation is induced anteriorly (open arrow). (**E-F''**) Ectopic induction of Wg (red in E, E') can be detected in the *Dref*-depleted compartment (arrows in E, E'), whereas *dpp-*lacZ expression appears unchanged (red in **F-F'**). Genotypes: (A-B'') and (D-E'') *en-*Gal4 UAS-*GFP/+;* UAS-*Dref-*RNAi/+. (C-C'') *en-*Gal4 UAS-*GFP/+;* UAS-*Dref-*RNAi/*puc*-lacZ. (F-F'') *en-*Gal4 UAS-*GFP/*+; UAS-*Dref-*RNAi/*dpp*-lacZ. Posterior is right, dorsal upwards. The dashed line marks the A/P compartment boundary. Scale bars: 100 μm.

**Fig 8:**
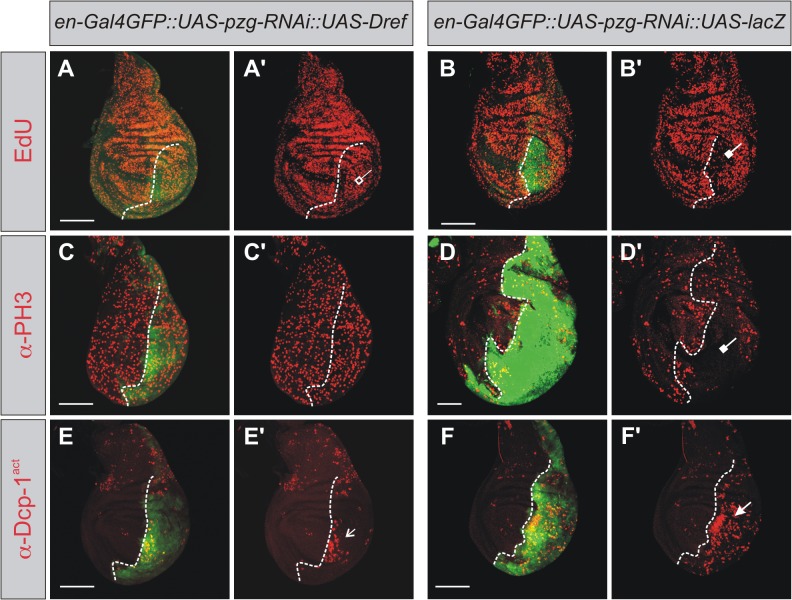
*Dref* overexpression ameliorates *pzg*-RNAi apoptosis defects. Comparison of the apoptotic consequences of *pzg* depletion in the presence (A-E’) or absence (B-F’) of ectopic *Dref* expression. (**A-D'**) Compared to the control (B,B',D,D'), overexpression of *Dref* in *pzg*-RNAi depleted cells ameliorates the cell cycle arrest defect (A,A',C,C'), visualized either with EdU (S-phase, red in A-B') or anti-PH3 (M-phase, red in C-D'). Repression of cell division by *pzg* depletion (repressive arrow in B', D'), is absent (C') or less pronounced in the *Dref* overexpression background (open repressive arrow in A'). Activation of Dcp-1 (anti-Dcp-1^act^, red in E-F') upon *pzg* downregulation (arrow in F'), is much weaker when *Dref* is overexpressed (open arrow in E'). Genotypes: (A, A'; C, C'; E, E') *en-*Gal4 UAS-*GFP UAS-pzg-*RNAi/+; UAS*-Dref/+*. (B, B'; D, D'; F, F') *en-*Gal4 UAS-*GFP UAS-pzg-*RNAi / UAS-*lacZ*. Posterior is right and dorsal up. The A/P compartment boundary is marked with a dashed line. Scale bars: 100 μm.

Apart from its role in N target gene activation, Pzg is important for cell proliferation as a member of the Trf2/Dref complex in *Drosophila* [40,74]. The transcription factor Dref regulates the expression of many proliferation-related genes, and has been described as a master key factor for cell proliferation (reviewed in [37]). We hence examined the consequences of *Dref*-RNAi depletion on apoptosis, AiP and AiA. To this end the UAS-*Dref*-RNAi line was used that has been shown before to specifically target *Dref* activity [75]. Inducing *Dref*-RNAi in the posterior half of the wing disc indeed triggered a strong apoptotic response, including robust Dcp1 caspase activation in the anterior half of late third instar wing discs (appr. 120 h AEL), indicative of AiA (Fig 7A-B''). Moreover, JNK signaling, i.e. *puc*-lacZ, was induced autonomously in the affected compartment, and to a lesser degree also in the anterior compartment of the wing disc (Fig 7C-C''). As expected, reduction of *Dref* activity was correlated with impaired cell proliferation as demonstrated by reduced EdU labeling in these areas (Fig 7D-D''). Proliferation rates were, however, significantly increased in the tissue abutting the *Dref* mutant cells (Fig 7D-D''). Moreover, similar to the effects of *pzg*-RNAi depletion, ectopic Wg induction was detected in the *Dref*-RNAi mutant tissue (Fig 7E-E''), whereas *dpp* activity appeared unaffected (Fig 7F-F''). As expected from our earlier work, Wg expression along the dorso-ventral boundary was not affected by the depletion of Dref, in support of the notion that the effects of Pzg on N regulation are Dref independent [40, 41]. Overall, induction of apoptosis with all its consequences is very similar upon the depletion of either Dref or Pzg during larval wing development. We therefore asked whether an increase of *Dref* activity might ameliorate the apoptotic consequences of *pzg* depletion. To this end we overexpressed UAS-*pzg*-RNAi together with UAS-*Dref* in the posterior wing disc compartment. To account for possible titration effects of Gal4 by the extra UAS-dose, we compared the effects of *Dref* overexpression with those obtained in a *pzg*-RNAi plus UAS-*lacZ* background. We observed a weaker apoptotic response in the UAS-lacZ background, (Fig 8). However, a concomitant increase of *Dref* activity ameliorated the *pzg*-RNAi proliferation defects much more robustly and strongly attenuated the induction of activated Dcp-1 caspase (Fig 8). Therefore, although Pzg and Dref are part of a large multi-subunit complex, increasing the amount of Dref protein helps to counterbalance the apoptotic effects resulting from *pzg* loss.

## Discussion

In this work we show that loss of *pzg* induces apoptosis, including the autonomous activation of *rpr* and of various Caspases, of JNK and of Dilp-8 expression. Moreover, we observed non-autonomous apoptosis induced proliferation (AiP) including the induction of Wingless as well as apoptosis induced apoptosis (AiA). Apparently, *pzg* is required for the survival of the cell which in its absence undergoes apoptosis with all its consequences.

Pzg is a nuclear protein found in different multimeric complexes including the Trf2/Dref complex and the NURF complex. The Pzg/NURF complex has been implied in the epigenetic regulation of N and EcR target genes [41,76], whereas Dref is involved in the regulation of replication and proliferation related genes (reviewed in [37]). Both, downregulation of *N* as well as of *Dref* induced apoptosis. Whereas the former was expected [51,72], the latter was not since overexpression rather than downregulation of *Dref* was associated with apoptosis so far [77,78], presumably because Dref overexpression is sufficient to drive terminally differentiated cells into a new cell cycle [78]. Very similar to *pzg* RNAi, downregulation of *Dref* not only resulted in autonomous JNK- and Caspase activation, but also induced ectopic expression of Wingless, which was not observed as a consequence of N depletion. Moreover, unlike the activation of N signaling activity, overexpression of *Dref* was sufficient to ameliorate the effects of *pzg* induced apoptosis. Overall we conclude that the apoptosis induced by a depletion of *pzg* is primarily triggered by a disturbance of the Pzg/Trf2/Dref complex. In this case, at least part of the apoptotic consequences may result from a dysregulatin of Pzg/Dref target genes involved in cell survival or cell death. For example, the failure to activate anti-apoptotic factors in the *pzg*- and *Dref*-RNAi mutant background might directly induce apoptosis. Amongst the plethora of Dref target genes is the *Drosophila* proto-oncogene *raf* [79]. Indeed it was shown that overexpression of Dref stimulates MAPK signaling activity [46]. MAPK, however, is an important negative regulator of pro-apoptotic gene activity in *Drosophila* [55,80], easily explaining the pro-apoptotic effects of a *Dref* loss. Moreover, inspection of the promoter region of the *Drosophila* inhibitor of apoptosis (*DIAP1*) gene reveals the presence of several potential DRE sites (S4 Fig). Hence, Dref may support cell survival by activating *DIAP1* under normal circumstances. Future will show whether Dref acts together with Pzg, i.e. whether Pzg binds to the promoters of cell death regulators as well. Dref, however, not only stimulates the expression of anti-apoptotic or survival factors, but it also promotes the transcriptional activation of the apoptosis inducer *dmp53* [81]. Similar to the apparent disparate role of Dref in the regulation of apoptosis, Dref not only promotes cell proliferation by the transcriptional activation of proliferation related genes like Pol alpha or PCNA, but Dref also inhibits proliferation as different members of the Hippo signaling pathway are Dref target genes as well [82,83].

Alternatively to a direct role of Dref and Pzg in the transcriptional regulation of genes involved in cell survival, cell death may arise as a consequence of conflicting signals that result from a collective dysregulation of the numerous Dref target genes. Depletion of *pzg* or *Dref* presumably affects a wide range of Dref-target genes. Dref target sequences have been found in more than 200 *Drosophila* genes, including replication and proliferation related genes, genes involved in growth, development and differentiation as well as components of protein biosynthesis or RNA binding proteins (reviewed in [37]; [84]). Therefore, it appears likely that crippling *pzg* activity, impairs the Dref-mediated cellular homeostasis, and hence the balance between survival and death decisions.

Recent molecular studies in *Drosophila* demonstrated that Pzg protein can be specifically detected at the telomeres of the chromosomes [85]. In *Drosophila*, the maintenance of telomeres is realized by a repeated transposition of retrotransposons instead of the telomerase-dependent extension of other eukaryotes (reviewed in [86,87]). Despite this difference, mutations that cause dysfunctions of the telomeres give rise to chromosome- and DNA-damage in all eukaryotes, and consequently result in apoptosis and in an increased lethality ([88,89]; reviewed in [90]). *Pzg* mutant animals were shown to suffer from moderate telomere instability, inferred from a significant increase in the in vivo incidence of telomere fusions in anaphase neuroblasts. Telomere fusions were attributed to major chromatin changes causing altered transcriptional activity of the retrotransposon Het-A due to the loss of *pzg* activity [91]. In accordance with a functional relationship between Pzg and Dref in this developmental context, *Dref* mutants show similar alterations in retrotransposon expression [92]. Moreover, specific Dref target sequences were identified in the promoters of several retrotransposons in *Drosophila*, implying a direct regulatory function of Dref on the retrotransposon activity and telomere elongation [92]. Therefore, apoptosis induction observed in *pzg* and *Dref* mutant tissues might involve excessive retrotransposon activity, destroying the fine-tuned genomic stability.

## Supporting Information

S1 Fig Quantification of eye sizes. Eye size of flies was determined in five females of each combination shown in Fig 1. Average eye area is shown in each column. The ordinate shows the percentage of eye area relative to the respective control (left column each, light grey, 100%). Error bars denote standard deviation. ***p<0.001; **p<0.01; *p<0.05; ns: not significant according to Student's T-test.
(DOC)

S2 Fig Induction of *pzg*-RNAi with *omb*-Gal4 provokes cell death. *pzg-*RNAi application in the most central part of the wing disc with *omb*-Gal4 induces *rpr-*lacZ (red in A, A', arrows), activated Drice (red in C, C', arrows), activated Caspase 3 (red in D, D', arrow), Dcp-1^act^ (red in E, E', arrows*)* and *puc*-lacZ (red in F, F', arrows). In contrast, the level of the anti-apoptotic protein DIAP1 is reduced (red in B, B', repressive arrows). (**A-A'''**) *omb-*Gal4*;* UAS*-pzg-*RNAi/*+*; *rpr-*lacZ*/+*, (**B-E''**) *omb-*Gal4*;* UAS*-pzg-*RNAi/*+*, (**F-F''**) *omb-*Gal4*;* UAS*-pzg-*RNAi/*+*; *puc-*lacZ*/+*. Anti-Putzig staining is shown in green. Posterior is right and dorsal up. The affected area is outlined. Scale bars: 100 μm.
(DOC)

S3 Fig Reduced *H* activity still induces apoptotic effects in *pzg*-RNAi mutant cells. Reducing the activity of the N repressor *Hairless* (*H*) formally enhances N activity but does not rescue the apoptotic consequences observed in *pzg*-RNAi mutant cells. (**A-B''**) Autonomous induction of Dcp-1^act^ (red in A, A', arrow) can be detected in wing discs app. 96 h AEL, whereas additional non autonomous Dcp-1^act^ activity is provoked in later stages (B, B', open arrows). (**C-D''**) Cell cycle progression is still autonomously impeded in *pzg*-RNAi depleted cells (cells in S-phase marked with EdU-labeling red in C, C', repressive arrow and cells in M-phase depicted with anti-PH3, red in D, D' repressive arrow). Enhanced proliferation in cells directly abutting the posterior compartment is still observed (open arrows in C' and D'). Anti-H staining is shown in blue (A'', B'', C'', D'') depicting loss of H protein by induction of *H*-RNAi. Posterior is right and dorsal up. The dashed line assigns the A/P compartment boundary. Scale bars: 100 μm.
(DOC)

S4 Fig Potential DRE sites in *Diap1*. According to flybase (R6.03; FB2014_06, released November 12th, 2014), there are 6 strongly supported transcripts *Diap1* RA-RF, transcribed from 5 different promoters (http://flybase.org/cgi-bin/gbrowse2/dmel/?Search=1;name=FBgn0260635). Potential DRE sites are marked with arrows and listed below. Transcript RB starts only 363 bp downstream of RF; they may share the DRE sites. No DRE sites were found in the proximity of the RA/RE transcription start. Dref regulation of RC appears less likely due to sequence divergence and distance of DRE.
(DOC)

## References

[1] Thompson CB. Apoptosis in the pathogenesis and treatment of disease. Science 1995; 267: 1456–1462. 7878464 10.1126/science.7878464

[2] Jacobson MD. Apoptosis: Bcl-2-related proteins get connected. Curr Biol. 1997; 7: R277–R281. 9115386 10.1016/s0960-9822(06)00136-9

[3] Hanahan D, Weinberg RA. The hallmarks of cancer. Cell 2000; 100: 57–70. 10647931 10.1016/s0092-8674(00)81683-9

[4] McIlwain DR, Berger T, Mak TW. Caspase functions in cell death and disease. Cold Spring Harb Perspect Biol. 2013; 5:a008656 10.1101/cshperspect.a008656 23545416 PMC3683896

[5] Kumar S. Caspase function in programmed cell death. Cell Death Differ. 2007; 14: 32–43. 17082813 10.1038/sj.cdd.4402060

[6] Berthelet J, Dubrez L. Regulation of apoptosis by inhibitors of apoptosis (IAPs). Cells 2013; 2: 163–187. 10.3390/cells2010163 24709650 PMC3972657

[7] White K, Grether ME, Abrams JM, Young L, Farrell K, Steller H. Genetic control of programmed cell death in *Drosophila* . Science 1994; 264: 677–683. 8171319 10.1126/science.8171319

[8] Grether ME, Abrams JM, Aqapite J, White K, Steller H. The *head involution defective* gene of *Drosophila melanogaster* functions in programmed cell death. Genes Dev. 1995; 9: 1694–1708. 7622034 10.1101/gad.9.14.1694

[9] Chen P, Nordstrom W, Gish B, Abrams JM. *grim*, a novel cell death gene in *Drosophila* . Genes Dev. 1996; 10: 1773–1782. 8698237 10.1101/gad.10.14.1773

[10] Wang SL, Hawkins CJ, Yoo SJ, Müller HA, Hay BA. The *Drosophila* caspase DIAP1 is essential for cell survival and is negatively regulated by HID. Cell 1999; 98: 453–463. 10481910 10.1016/s0092-8674(00)81974-1

[11] Goyal L, McCall K, Agapite J, Hartwieg E, Steller H. Induction of apoptosis by *Drosophila reaper*, *hid* and *grim* through inhibition of IAP function. EMBO J. 2000; 19: 589–597. 10675328 10.1093/emboj/19.4.589 PMC305597

[12] Ryoo HD, Bergmann A, Gonen H, Ciechanover A, Steller H. Regulation of *Drosophila* IAP1 degradation and apoptosis by *reaper* and *ubcD1* . Nature Cell Biol. 2002; 4: 432–438. 12021769 10.1038/ncb795

[13] Yoo SJ, Huh JR, Muro I, Yu H, Wang L, Wang SL et al Hid, Rpr and Grim negatively regulate DIAP1 levels through distinct mechanisms. Nature Cell Biol. 2002; 6: 416–424. 12021767 10.1038/ncb793

[14] Fan Y, Bergmann A. Apoptosis-induced compensatory proliferation. The cell is dead: Long live the cell! Trends Cell Biol. 2008; 18: 467–473. 10.1016/j.tcb.2008.08.001 18774295 PMC2705980

[15] Bergmann A, Steller H. Apoptosis, stem cells, and tissue regeneration. Sci Signal 2010; 3(145):re8 10.1126/scisignal.3145re8 20978240 PMC2991142

[16] Morata G, Shlevkov E, Pérez-Garijo A. Mitogenic signaling from apoptotic cells in *Drosophila* . Develop Growth Differ. 2011; 53: 168–176. 10.1111/j.1440-169X.2010.01225.x 21338343

[17] Ryoo HD, Bergmann A. The role of apoptosis-induced proliferation for regeneration and cancer. Cold Spring Harb Perspect Biol. 2012; 4: a008797 10.1101/cshperspect.a008797 22855725 PMC3405855

[18] Pérez-Garijo A, Martín FA, Morata G. Caspase inhibition during apoptosis causes abnormal signalling and developmental aberrations in *Drosophila* . Development 2004; 131: 5591–5598. 15496444 10.1242/dev.01432

[19] Ryoo HD, Gorenc T, Steller H. Apoptotic cells can induce compensatory proliferation through the JNK and the Wingless signaling pathways. Dev Cell 2004; 7: 491–501. 15469838 10.1016/j.devcel.2004.08.019

[20] Vidal M, Cagan RL. *Drosophila* models for cancer research. Curr Opin Genet Dev. 2006; 16: 10–16. 16359857 10.1016/j.gde.2005.12.004

[21] Fan Y, Wang S, Hernandez J, Betul Venigun V, Hertlein G, Fogarty CE et al Genetic models of apoptosis-induced proliferation decipher activation of JNK and identify a requirement of EGFR signaling for tissue regenerative responses in *Drosophila* . PLOS Genet. 2014; 10: e1004131 10.1371/journal.pgen.1004131 24497843 PMC3907308

[22] Lawrence P, Struhl G. Morphogens, compartments, and pattern: lessons from *Drosophila*? Cell 1996; 85: 951–961. 8674123 10.1016/s0092-8674(00)81297-0

[23] Tabata T, Takei Y. Morphogens, their identification and regulation. Development 2004; 131: 703–712. 14757636 10.1242/dev.01043

[24] Clem RJ, Fechheimer M, Miller MK. Prevention of apoptosis by a baculovirus gene during infection of insect cells. Science 1991; 254: 1388–1390. 1962198 10.1126/science.1962198

[25] Kondo S, Senoo-Matsuda N, Hiromi Y, Miura M. DRONC coordinates cell death and compensatory proliferation. Mol Cell Biol. 2006; 26: 7258–7268. 16980627 10.1128/MCB.00183-06 PMC1592896

[26] Huh JR, Guo M, Hay BA. Compensatory proliferation induced by cell death in the *Drosophila* wing disc requires activity of the apical cell death caspase Dronc in a nonapoptotic role. Curr Biol. 2004; 14: 1262–1266. 15268856 10.1016/j.cub.2004.06.015

[27] Pérez-Garijo A, Shlevkov E, Morata G. The role of Dpp and Wg in compensatory proliferation and in the formation of hyperplastic overgrowths caused by apoptotic cells in the *Drosophila* wing disc. Development 2009; 136: 1169–1177. 10.1242/dev.034017 19244279

[28] Bergantiños C, Corominas M, Serras F. Cell death-induced regeneration in wing discs requires JNK signaling. Development 2010; 137: 1169–1179. 10.1242/dev.045559 20215351

[29] Worley MI, Setiawan L, Hariharan IK. Regeneration and transdetermination in *Drosophila* imaginal discs. Annu Rev Genet. 2012; 46: 289–310. 10.1146/annurev-genet-110711-155637 22934642

[30] Bosch M, Serras F, Martin-Blanco F, Baguna J. JNK signaling pathway required for wound healing in regenerating *Drosophila* wing imaginal discs. Dev Biol. 2005; 280: 73–86. 15766749 10.1016/j.ydbio.2005.01.002

[31] Mattila J, Omelyanchuck L, Kyttala S, Turunen H, Nokkala S. Role of Jun N-terminal kinase (JNK) signaling in the wound healing and regeneration of a *Drosophila melanogaster* wing imaginal disc. Int J Dev Biol. 2005; 49: 391–399. 15968584 10.1387/ijdb.052006jm

[32] Iagki T, Pagliarini RA, Xu T. Loss of cell polarity drives tumor growth and invasion through JNK activation in *Drosophila* . Curr Biol. 2006; 16: 1139–1146. 16753569 10.1016/j.cub.2006.04.042

[33] You H, Padmashali RM, Ranganathan A, Lei P, Girnius N, Davis RJ et al JNK regulates compliance-induced adherens junctions formation in epithelial cells and tissues. J Cell Sci. 2013; 126: 2718–2729. 10.1242/jcs.122903 23591817

[34] Pérez-Garijo A, Fuchs Y, Steller H. Apoptotic cells can induce non-autonomous apoptosis through the TNF pathway. eLife 2013; 2: e01004 10.7554/eLife.01004 24066226 PMC3779319

[35] Morata G, Herrera SC. Eiger triggers death from afar. eLife 2013; 2:e01388 10.7554/eLife.01388 24069529 PMC3780540

[36] Bray SJ. Notch signaling: a simple pathway becomes complex. Nat Rev Mol Cell Biol. 2006; 7: 678–689. 16921404 10.1038/nrm2009

[37] Matsukage A, Hirose F, Yoo MA, Yamaguchi M. The DRE/DREF transcriptional regulatory system: a master key for cell proliferation. Biochim Biophys Acta 2008; 1779: 81–90. 18155677 10.1016/j.bbagrm.2007.11.011

[38] Djiane A, Krejci A, Bernard F, Fexova S, Millen K, Bray SJ. Dissecting the mechanisms of Notch induced hyperplasia. EMBO J. 2013; 32: 60–71. 10.1038/emboj.2012.326 23232763 PMC3545308

[39] Dominguez M. Oncogenic programmes and Notch activity: An 'organized crime'? Semin Cell Dev Biol. 2014; 28: 78–85. 10.1016/j.semcdb.2014.04.012 24780858

[40] Kugler SJ, Nagel AC. *putzig* is required for cell proliferation and regulates Notch activity in *Drosophila* . Mol Biol Cell. 2007; 18: 3733–3740. 17634285 10.1091/mbc.E07-03-0263 PMC1995712

[41] Kugler SJ, Nagel AC. A novel Pzg-NURF complex regulates Notch target gene activity. Mol Biol Cell. 2010; 21: 3443–3448. 10.1091/mbc.E10-03-0212 20685964 PMC2947479

[42] Neufeld TP, Edgar BA. Connections between growth and the cell cycle. Curr Opin Cell Biol. 1998; 10: 784–790. 9914170 10.1016/s0955-0674(98)80122-1

[43] Hay BA, Maile R, Rubin GM. P element insertion-dependent gene activation in the *Drosophila* eye. Proc Natl Acad Sci USA 1997; 10: 5195–5200. 9144214 10.1073/pnas.94.10.5195 PMC24655

[44] White K, Tahaoglu E, Steller H. Cell killing by the *Drosophila* gene *reaper* . Science 1996; 271: 805–807. 8628996 10.1126/science.271.5250.805

[45] Lecuit T, Brook WJ, Ng M, Celleja M, Sun H, Cohen SM. Two distinct mechanisms for long-range patterning by Decapentaplegic in the *Drosophila* wing. Nature 1996; 381: 387–393. 8632795 10.1038/381387a0

[46] Yoshida H, Kwon E, Hirose F, Otsuki K, Yamada M, Yamaguchi M. DREF is required for EGFR signaling during *Drosophila* wing development. Genes Cells 2004; 9: 935–944. 15461664 10.1111/j.1365-2443.2004.00775.x

[47] Nagel AC, Krejci A, Tenin G, Bravo-Patiño A, Bray S, Maier D et al Hairless-mediated repression of Notch target genes requires the combined activity of Groucho and CtBP corepressors. Mol Cell Biol. 2005; 25: 10433–10441. 16287856 10.1128/MCB.25.23.10433-10441.2005 PMC1291231

[48] Blackman RK, Sanicola M, Raftery LA, Gillevet T, Gelbart WM. An extensive 3' cis-regulatory region directs the imaginal disk expression of *decapentaplegic*, a member of the TGF-beta family in *Drosophila* . Development 1991; 111: 657–666. 1908769 10.1242/dev.111.3.657

[49] Ring JM, Martinez-Arias A. *puckered*, a gene involved in position-specific cell differentiation in the dorsal epidermis of the *Drosophila* larva. Dev Suppl. 1993: 251–259. 8049480

[50] Sogame N, Kim M, Abrams JM. *Drosophila* p53 preserves genomic stability by regulating cell death. Proc Natl Acad Sci USA 2003; 100: 4696–4701. 12672954 10.1073/pnas.0736384100 PMC153618

[51] Müller D, Kugler SJ, Preiss A, Maier D, Nagel AC. Genetic modifier screens on Hairless gain-of-function phenotypes reveal genes involved in cell differentiation, cell growth and apoptosis in *Drosophila melanogaster* . Genetics 2005; 171: 1137–1152. 16118195 10.1534/genetics.105.044453 PMC1456817

[52] Maier D, Nagel AC, Preiss A. Two isoforms of the Notch antagonist Hairless are produced by differential translation initiation. Proc Natl Acad Sci USA 2002; 99: 15480–15485. 12422020 10.1073/pnas.242596699 PMC137742

[53] Colombani J, Andersen DS, Léopold P. Secreted peptide Dilp8 coordinates *Drosophila* tissue growth with developmental timing. Science 2012; 336: 582–585. 10.1126/science.1216689 22556251

[54] McCall K, Steller H. Facing death in the fly: genetic analysis of apoptosis in *Drosophila* . Trends Genet. 1997; 13: 222–226. 9196327 10.1016/S0168-9525(97)01126-8

[55] Bergmann A, Agapite J, McCall K, Steller H. The *Drosophila* gene *hid* is a direct molecular target of Ras-dependent survival signaling. Cell 1998; 95: 331–341. 9814704 10.1016/s0092-8674(00)81765-1

[56] Abrams JM. An emerging blueprint for apoptosis in *Drosophila* . Trends Cell Biol. 1999; 9: 435–440. 10511707 10.1016/s0962-8924(99)01646-3

[57] Martin SJ. Destabilizing influences in apoptosis: sowing the seeds of IAP destruction. Cell 2002; 109: 793–796. 12110175 10.1016/s0092-8674(02)00802-4

[58] Richardson H, Kumar S. Death to flies: *Drosophila* as a model system to study programmed cell death. J Immunol Methods 2002; 265: 21–38. 12072176 10.1016/s0022-1759(02)00068-6

[59] Milán M, Campuzano S, Garcia-Bellido A. Developmental parameters of cell death in the wing disc of *Drosophila* . Proc Natl Acad Sci USA 1997; 94: 5691–5696. 9159134 10.1073/pnas.94.11.5691 PMC20840

[60] Dhanasekaran DN, Reddy EP. JNK signaling in apoptosis. Oncogene 2008; 27: 6245–6251. 10.1038/onc.2008.301 18931691 PMC3063296

[61] Igaki T. Correcting developmental errors by apoptosis: lessons from Drosophila JNK signaling. Apoptosis 2009; 14: 1021–1028. 10.1007/s10495-009-0361-7 19466550

[62] Martin-Blanco E, Gampel A, Ring J, Virdee K, Kirov N, Tolkovsky AM et al *puckered* encodes a phosphatase that mediates a feedback loop regulating JNK activity during dorsal closure in *Drosophila* . Genes Dev. 1998; 12: 557–570. 9472024 10.1101/gad.12.4.557 PMC316530

[63] Garelli A, Gontijo AM, Miguela V, Caparros E, Dominguez M. Imaginal discs secrete insulin-like peptide 8 to mediate plasticity of growth and maturation. Science 2012; 336: 579–582. 10.1126/science.1216735 22556250

[64] Smith-Bolton RK, Worley MI, Kanda H, Hariharan IK. Regenerative growth in *Drosophila* imaginal discs is regulated by Wingless and Myc. Dev Cell 2009; 16: 797–809. 10.1016/j.devcel.2009.04.015 19531351 PMC2705171

[65] Herrera SC, Martin R, Morata G. Tissue homeostasis in the wing disc of *Drosophila melanogaster*: immediate response to massive damage during development. PLOS Genet. 2013; 9: e1003446 10.1371/journal.pgen.1003446 23633961 PMC3636033

[66] Kashio S, Obata F, Miura M. Interplay of cell proliferation and cell death in *Drosophila* tissue regeneration. Develop Growth Differ. 2014; 56: 368–375. 10.1111/dgd.12139 24819984

[67] Brancolini C, Lazarevic D, Rodriguez J, Schneider C. 2005 Dismanteling cell-cell contacts during apoptosis is coupled to a caspase-dependent proteolytic cleavage of beta-catenin. J Cell Biol. 2005; 139: 759–771. 10.1083/jcb.139.3.759 PMC2141701 9348292

[68] Kessler T, Müller AJ. Cleavage of Armadillo/beta catenin by the Caspase DrICE in *Drosophila* apoptotic epithelial cells. BMC Dev Biol. 2009; 9: 15 10.1186/1471-213X-9-15 19232093 PMC2657781

[69] Martín F, Peréz-Garijo A, Morata G. Apoptosis in *Drosophila*: compensatory proliferation and undead cells. Int J Dev Biol. 2009; 53: 1341–1347. 10.1387/ijdb.072447fm 19247932

[70] Mollereau B, Pérez-Garijo A, Bergmann A, Miura N, Gerlitz O, Ryoo HD et al Compensatory proliferation and apoptosis-induced proliferation: a need for clarification. Cell Death Differ. 2013; 20: 181 10.1038/cdd.2012.82 22722336 PMC3524636

[71] Hay BA, Wolff T, Rubin GM. Expression of baculovirus p35 prevents cell death in *Drosophila* . Development 1994; 120: 2121–2129. 7925015 10.1242/dev.120.8.2121

[72] Protzer CE, Wech I, Nagel AC. Hairless induces cell death by downregulation of EGFR signaling activity. J Cell Sci. 2008; 121: 3167–3176. 10.1242/jcs.035014 18765565

[73] Djiane A, Zaessinger S, Babaoglan AB, Bray SJ. Notch inhibits yorkie activity in *Drosophila* wing discs. PLOS One 2014; 9: e106211 10.1371/journal.pone.0106211 25157415 PMC4144958

[74] Hochheimer A, Zhou S, Zheng S, Holmes MC, Tjian R. TRF2 associates with DREF and directs promoter-selective gene expression in *Drosophila* . Nature 2002; 420: 439–445. 12459787 10.1038/nature01167

[75] Iyer EPR, Iyer SC, Sullivan L, Wang D, Meduri R, Graybeal LL et al Functional genomic analyses of two morphologically distinct classes of *Drosophila* sensory neurons: Post-mitotic roles of transcription factors in dendritic patterning. PLOS One 2013; 8: e72434 10.1371/journal.pone.0072434 23977298 PMC3744488

[76] Kugler SJ, Gehring E-M, Wallkamm V, Krüger V, Nagel AC. The Putzig-NURF nucleosome remodeling complex is required for ecdysone receptor signaling and innate immunity in *Drosophila melanogaster* . Genetics 2011; 188: 127–139. 10.1534/genetics.111.127795 21385730 PMC3120143

[77] Hirose F, Ohshima N, Shiraki M, Inoue YH, Taguchi O, Nishi Y et al Ectopic expression of DREF induces DNA synthesis, apoptosis, and unusual morphogenesis in the *Drosophila* eye imaginal disc: possible interaction with Polycomb and trithorax group proteins. Mol Cell Biol 2001; 21: 7231–7242. 11585906 10.1128/MCB.21.21.7231-7242.2001 PMC99898

[78] Hyun J, Jasper H, Bohmann D. DREF is required for efficient growth and cell cycle progression in *Drosophila* imaginal discs. Mol Cell Biol. 2005; 25: 5590–5598. 15964814 10.1128/MCB.25.13.5590-5598.2005 PMC1157005

[79] Ryu JR, Choi TY, Kwon EJ, Lee WH, Nishida Y, Hayashi Y et al Transcriptional regulation of the *Drosophila raf* proto-oncogene by the DNA replication-related element (DRE)/DRE-binding factor (DREF) system. Nucleic Acids Res. 1997; 25: 794–799. 9016631 10.1093/nar/25.4.794 PMC146497

[80] Kurada P, White K. Ras promotes cell survival in *Drosophila* by downregulating hid expression. Cell 1998; 95: 319–329. 9814703 10.1016/s0092-8674(00)81764-x

[81] Trong-Tue N, Thao DT, Yamaguchi M. Role of DREF in transcriptional regulation of the *Drosophila p53* gene. Oncogene 2010; 29: 2060–9. 10.1038/onc.2009.483 20101238

[82] Fujiwara S, Ida H, Yoshioka Y, Yoshida H, Yamaguchi M. The *warts* gene as a novel target of the *Drosophila* DRE/DREF transcription pathway. Am J Cancer Res. 2012; 2: 36–44. 22206044 PMC3236570

[83] Vo N, Horii T, Yanai H, Yoshida H, Yamaguchi M. The Hippo pathway as a target of the *Drosophila* DRE/DREF transcriptional regulatory pathway. Sci Rep. 2014; 4:7196 10.1038/srep07196 25424907 PMC4244634

[84] Ohler U, Liao G-C, Niemann H, Rubin GM. Computational analysis of core promoters in the *Drosophila* genome. Genome Biology 2002; 3 (12): research0087.1–0087.12. 10.1186/gb-2002-3-12-research0087 PMC151189 12537576

[85] Andreyeva EN, Belyaeva ES, Semeshin VF, Pokholkova GV, Zhimulev IF. Three distinct chromatin domains in telomere ends of polytene chromosomes in *Drosophila melanogaster Tel* mutants. J Cell Sci. 2005; 118: 5465–5477. 16278293 10.1242/jcs.02654

[86] Stewart SA, Weinberg RA. Telomeres: cancer to human aging. Ann Rev Cell Dev Biol. 2006; 22: 531–557. 16824017 10.1146/annurev.cellbio.22.010305.104518

[87] Mason JM, Frydrychova RC, Biessmann H. *Drosophila* telomeres: an exception providing new insights. BioEssays 2008; 30: 25–37. 18081009 10.1002/bies.20688 PMC2804870

[88] Ahmad K, Golic KG. Telomere loss in somatic cells of *Drosophila* causes cell cycle arrest and apoptosis. Genetics 1999; 151: 1041–1051. 10049921 10.1093/genetics/151.3.1041 PMC1460522

[89] Titen SWA, Golic KG. Telomere loss provokes multiple pathways to apoptosis and produces genomic instability in *Drosophila melanogaster* . Genetics 2008; 180: 1821–1832. 10.1534/genetics.108.093625 18845846 PMC2600924

[90] Nigg EA. Genome instability in cancer development In: Advances in experimental Medicine and Biology. Springer, New York 2005

[91] Silva-Sousa R, López-Panadès E, Piñeyro D, Casacuberta E. The chromosomal proteins JIL-1 and Z4/Putzig regulate the telomeric chromatin in *Drosophila melanogaster* . PLOS Genet. 2012; 8: e1003153 10.1371/journal.pgen.1003153 23271984 PMC3521665

[92] Silva-Sousa R, Varela MD, Casacuberta E. The Putzig partners Dref, Trf2 and Ken are involved in the regulation of the *Drosophila* telomere retrotransposons, Het-A and TART. Mob DNA 2013; 4: 18 10.1186/1759-8753-4-18 23822164 PMC3726405

