# Supplementary material for: Loss of putzig Activity Results in Apoptosis during Wing Imaginal Development in Drosophila
Source: PLoS One. 2015 Apr 20;10(4):e0124652. doi: 10.1371/journal.pone.0124652 (PMC4403878; doi:10.1371/journal.pone.0124652)
Supplement: S1 Fig — Eye size of flies was determined in five females of each combination shown in Fig 1. Average eye area is shown in each column. The ordinate shows the percentage of eye area relative to the respective control (left column each, light grey, 100%). Error bars denote standard deviation. ***p<0.001; **p<0.01; *p<0.05; ns: not significant according to Student's T-test. (DOC) [file pone.0124652.s001.doc]

**
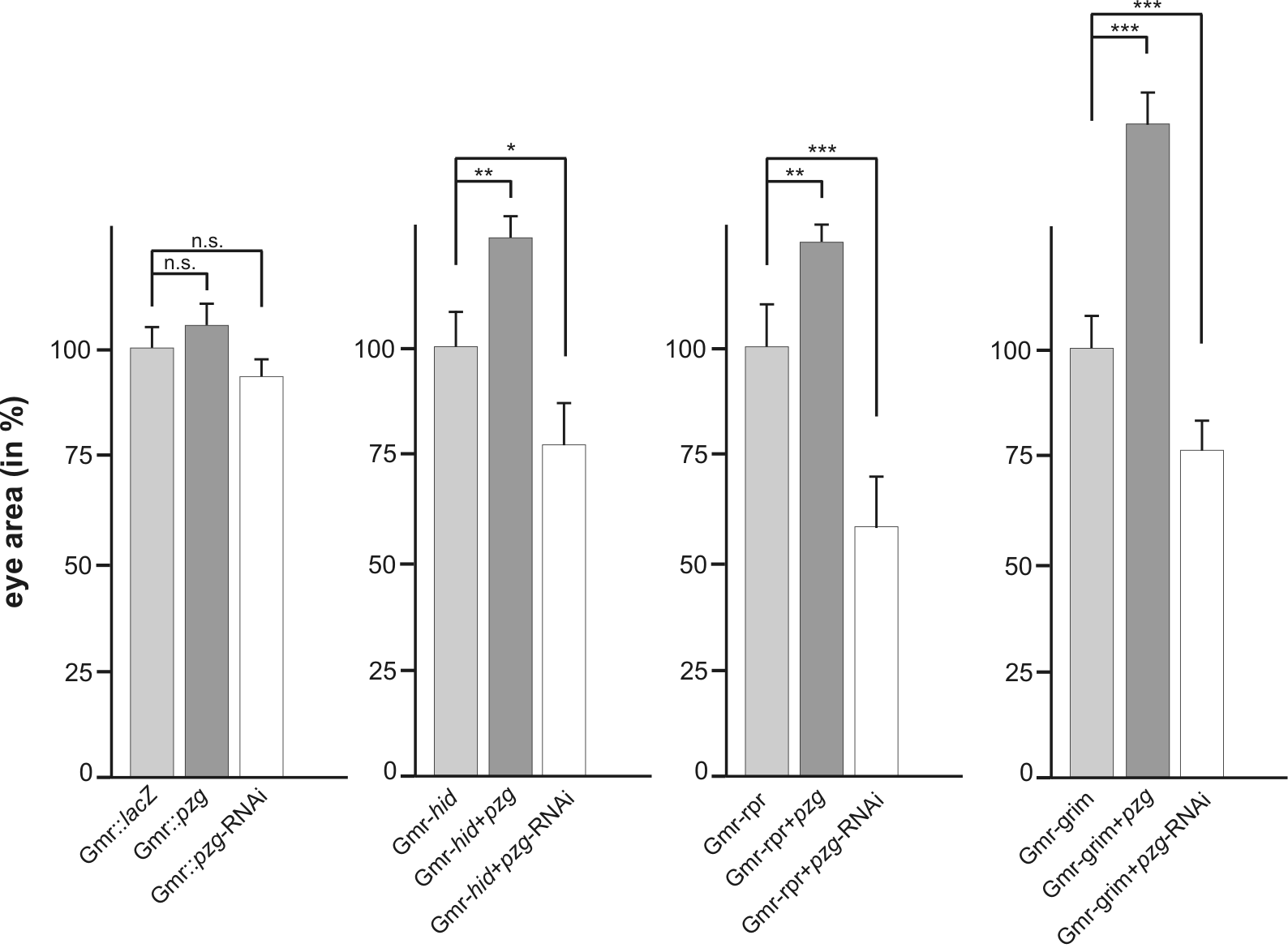
**

**S1 Fig. Quantification of eye sizes**

Eye size of flies was determined in five females of each combination shown in Fig.1. Average eye area is shown in each column. The ordinate shows the percentage of eye area relative to the respective control (left column each, light grey, 100%). Error bars denote standard deviation. ***p<0.001; **p<0.01; *p<0.05; ns: not significant according to Student's T-test.
