# Supplementary material for: Loss of putzig Activity Results in Apoptosis during Wing Imaginal Development in Drosophila
Source: PLoS One. 2015 Apr 20;10(4):e0124652. doi: 10.1371/journal.pone.0124652 (PMC4403878; doi:10.1371/journal.pone.0124652)
Supplement: S2 Fig — pzg-RNAi application in the most central part of the wing disc with omb-Gal4 induces rpr-lacZ (red in A, A', arrows), activated Drice (red in C, C', arrows), activated Caspase 3 (red in D, D', arrow), Dcp-1act (red in E, E', arrows) and puc-lacZ (red in F, F', arrows). In contrast, the level of the anti-apoptotic protein DIAP1 is reduced (red in B, B', repressive arrows). (A-A''') omb-Gal4; UAS-pzg-RNAi/+; rpr-lacZ/+, (B-E'') omb-Gal4; UAS-pzg-RNAi/+, (F-F'') omb-Gal4; UAS-pzg-RNAi/+; puc-lacZ/+. Anti-Putzig staining is shown in green. Posterior is right and dorsal up. The affected area is outlined. Scale bars: 100 μm. (DOC) [file pone.0124652.s002.doc]

**
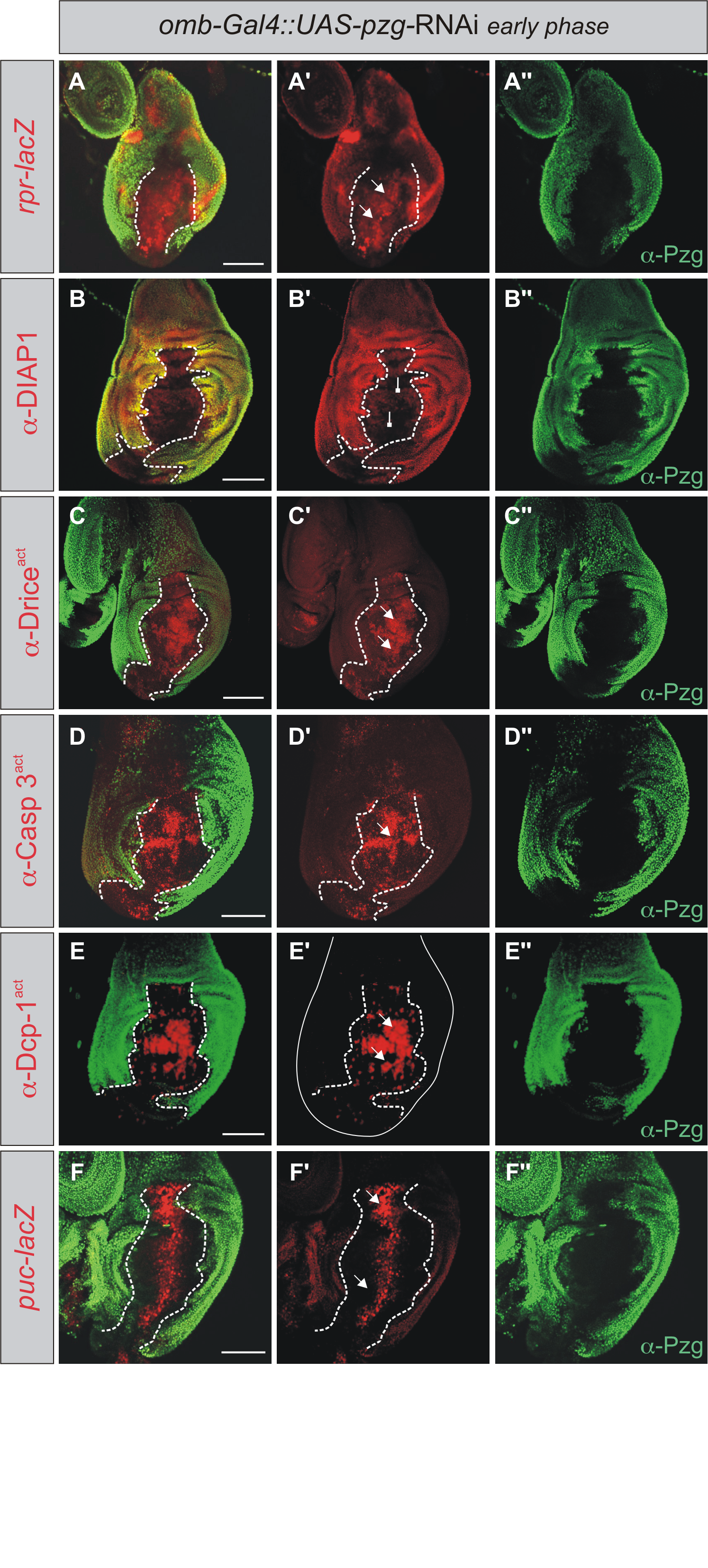
**

**S2 Fig. Induction of *pzg*-RNAi with *omb*-Gal4 provokes cell death**

*pzg-*RNAi application in the most central part of the wing disc with *omb*-Gal4 induces *rpr-*lacZ (red in A, A', arrows), activated Drice (red in C, C', arrows), activated Caspase 3 (red in D, D', arrow), Dcp-1^act^ (red in E, E', arrows*)* and *puc*-lacZ (red in F, F', arrows). In contrast, the level of the anti-apoptotic protein DIAP1 is reduced (red in B, B', repressive arrows). (**A-A'''**) *omb-*Gal4*;* UAS*-pzg-*RNAi/*+*; *rpr-*lacZ*/+,* (**B-E''**) *omb-*Gal4*;* UAS*-pzg-*RNAi/*+*, (**F-F''**) *omb-*Gal4*;* UAS*-pzg-*RNAi/*+*; *puc-*lacZ*/+*. Anti-Putzig staining is shown in green. Posterior is right and dorsal up. The affected area is outlined. Scale bars: 100 µm.
