# Supplementary material for: Loss of putzig Activity Results in Apoptosis during Wing Imaginal Development in Drosophila
Source: PLoS One. 2015 Apr 20;10(4):e0124652. doi: 10.1371/journal.pone.0124652 (PMC4403878; doi:10.1371/journal.pone.0124652)
Supplement: S3 Fig — Reducing the activity of the N repressor Hairless (H) formally enhances N activity but does not rescue the apoptotic consequences observed in pzg-RNAi mutant cells. (A-B'') Autonomous induction of Dcp-1act (red in A, A', arrow) can be detected in wing discs app. 96 h AEL, whereas additional non autonomous Dcp-1act activity is provoked in later stages (B, B', open arrows). (C-D'') Cell cycle progression is still autonomously impeded in pzg-RNAi depleted cells (cells in S-phase marked with EdU-labeling red in C, C', repressive arrow and cells in M-phase depicted with anti-PH3, red in D, D' repressive arrow). Enhanced proliferation in cells directly abutting the posterior compartment is still observed (open arrows in C' and D'). Anti-H staining is shown in blue (A'', B'', C'', D'') depicting loss of H protein by induction of H-RNAi. Posterior is right and dorsal up. The dashed line assigns the A/P compartment boundary. Scale bars: 100 μm. (DOC) [file pone.0124652.s003.doc]

**
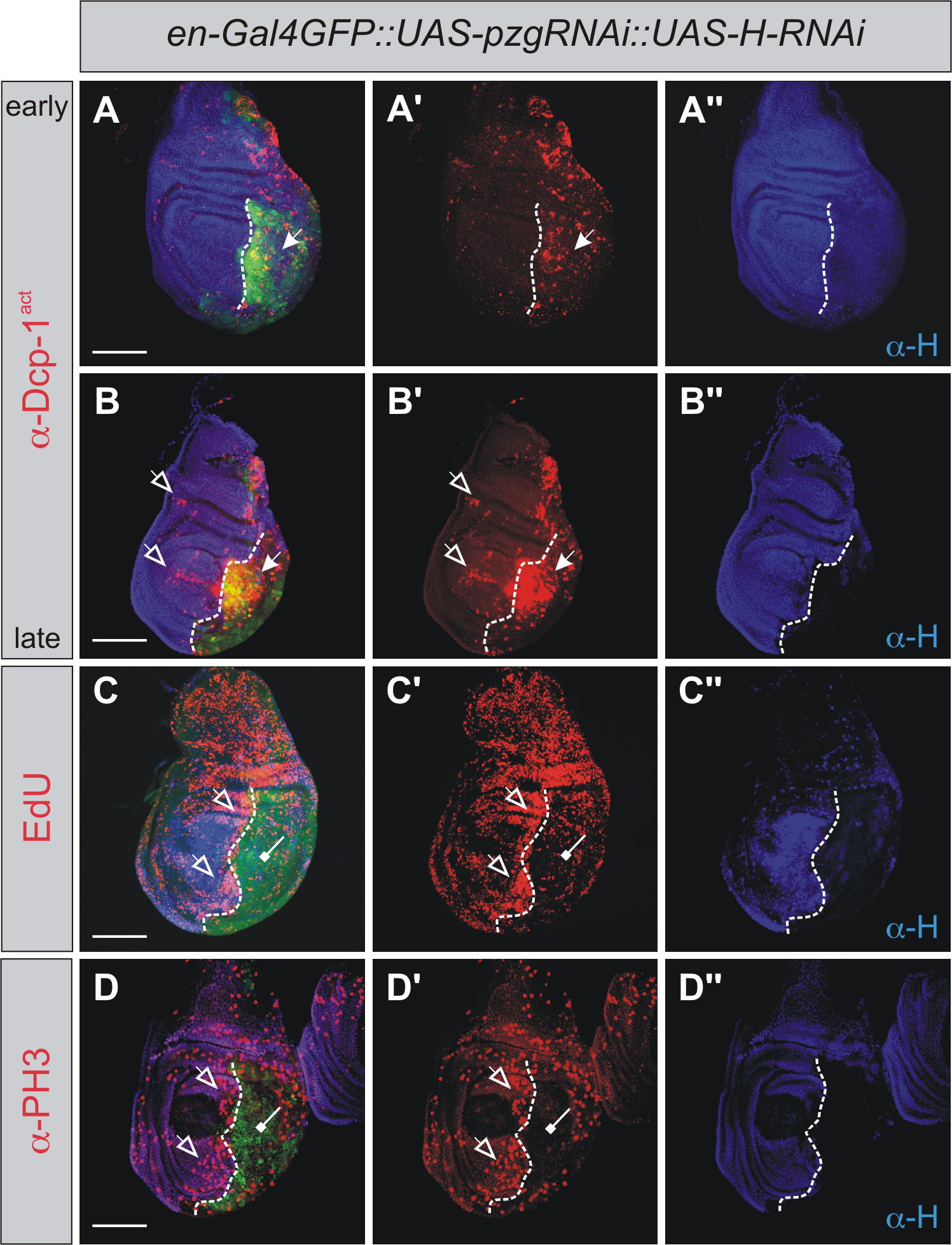
**

**S3 Fig. Reduced *H* activity still induces apoptotic effects in *pzg*-RNAi mutant cells**

Reducing the activity of the N repressor *Hairless* (*H*) formally enhances N activity but does not rescue the apoptotic consequences observed in *pzg*-RNAi mutant cells. (**A-B''**) Autonomous induction of Dcp-1^act^ (red in A, A', arrow) can be detected in wing discs app. 96 h AEL, whereas additional non autonomous Dcp-1^act^ activity is provoked in later stages (B, B', open arrows). (**C-D''**) Cell cycle progression is still autonomously impeded in *pzg*-RNAi depleted cells (cells in S-phase marked with EdU-labeling red in C, C', repressive arrow and cells in M-phase depicted with anti-PH3, red in D, D' repressive arrow). Enhanced proliferation in cells directly abutting the posterior compartment is still observed (open arrows in C' and D'). Anti-H staining is shown in blue (A'', B'', C'', D'') depicting loss of H protein by induction of *H*-RNAi. Posterior is right and dorsal up. The dashed line assigns the A/P compartment boundary. Scale bars: 100 µm.
