# Supplementary material for: Loss of putzig Activity Results in Apoptosis during Wing Imaginal Development in Drosophila
Source: PLoS One. 2015 Apr 20;10(4):e0124652. doi: 10.1371/journal.pone.0124652 (PMC4403878; doi:10.1371/journal.pone.0124652)
Supplement: S4 Fig — According to flybase (R6.03; FB2014_06, released November 12th, 2014), there are 6 strongly supported transcripts Diap1 RA-RF, transcribed from 5 different promoters (http://flybase.org/cgi-bin/gbrowse2/dmel/?Search=1;name=FBgn0260635). Potential DRE sites are marked with arrows and listed below. Transcript RB starts only 363 bp downstream of RF; they may share the DRE sites. No DRE sites were found in the proximity of the RA/RE transcription start. Dref regulation of RC appears less likely due to sequence divergence and distance of DRE. (DOC) [file pone.0124652.s004.doc]

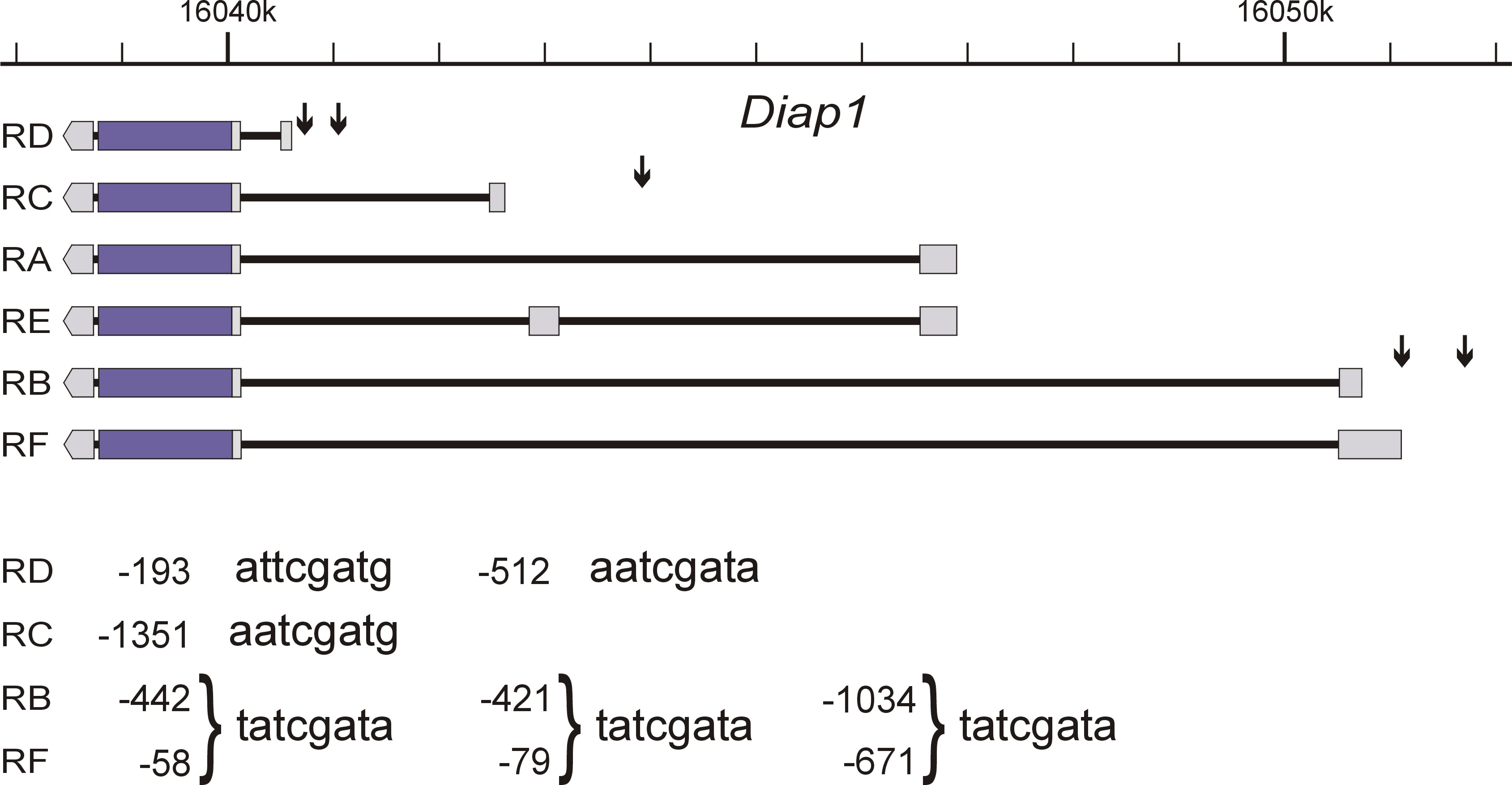


**S4 Fig. Potential DRE sites in *Diap1***

According to flybase (R6.03; FB2014_06, released November 12th, 2014), there are 6 strongly supported transcripts *Diap1* RA-RF, transcribed from 5 different promoters (http://flybase.org/cgi-bin/gbrowse2/dmel/?Search=1;name=FBgn0260635). Potential DRE sites are marked with arrows and listed below. Transcript RB starts only 363 bp downstream of RF; they may share the DRE sites. No DRE sites were found in the proximity of the RA/RE transcription start. Dref regulation of RC appears less likely due to sequence divergence and distance of DRE.
